# Lignin from Plant-Based Agro-Industrial Biowastes: From Extraction to Sustainable Applications

**DOI:** 10.3390/polym17070952

**Published:** 2025-03-31

**Authors:** Soledad Mateo, Giacomo Fabbrizi, Alberto J. Moya

**Affiliations:** 1 Chemical, Environmental and Materials Department, University of Jaén, Campus Las Lagunillas, 23071 Jaén, Spain; smateo@ujaen.es; 2 Olive Grove and Olive Oil Research Institute, 23071 Jaén, Spain; 3 Department of Chemistry, Biology and Biotechnology, Università degli Studi di Perugia, 06122 Perugia, Italy; giacomo.fabbrizi@unipg.it; 4 CIRIAF-CRB (Biomass Research Centre), Department of Engineering, Università degli Studi di Perugia, Via G. Duranti, 67, 06125 Perugia, Italy

**Keywords:** lignin, lignocellulosic agro-industrial wastes, treatments, delignification, applications

## Abstract

Lignin, the most abundant aromatic polymer in nature, plays a critical role in lignocellulosic biomasses by providing structural support. However, its presence complicates the industrial exploitation of these materials for biofuels, paper production and other high-value compounds. Annually, the industrial extraction of lignin reaches an estimated 225 million tons, yet only a fraction is recovered for reuse, with most incinerated as low-value fuel. The growing interest in lignin potential has sparked research into sustainable recovery methods from lignocellulosic agro-industrial wastes. This review examines the chemical, physical and physicochemical processes for isolating lignin, focusing on innovative, sustainable technologies that align with the principles of a circular economy. Key challenges include lignin structural complexity and heterogeneity, which hinder its efficient extraction and application. Nonetheless, its properties such as high thermal stability, biodegradability and abundant carbon content place lignin as a promising material for diverse industrial applications, including chemical synthesis and energy generation. A structured analysis of advancements in lignin extraction, characterization and valorization offers insights into transforming this undervalued by-product into a vital resource, reducing reliance on non-renewable materials while addressing environmental sustainability.

## 1. Introduction

Lignin is the most abundant aromatic polymer in nature [1] and it is naturally found as part of a wide variety of lignocellulosic materials. Lignin provides mechanical support, acting as a binder material for cellulose and hemicellulose fibers, two major polysaccharide fractions in this type of biomass. Lignin structure is dependent on the type of biomass and is also quite complex, due to its great aromaticity and high degree of branching and cross-linking. The presence of lignin can hinder the use of lignocellulosic biomass for the production of high value-added compounds, e.g., ethanol, xylitol, butanol and paper pulp. For these processes, lignin has to be removed in advance, since it causes strong inhibitory effects in processes for obtaining biofuels, reducing yields in both enzymatic hydrolysis and fermentation steps, or favoring the appearance of dark coloration in the cellulose pulp. This determines the existence of industrial processes in which there are waste flows with a high content of non-recovered lignin, having a negative impact on the environment. The annual extraction of lignin in industry is, in fact, in the range 1.5–1.8% of the total lignin present in the biosphere, and the industrial generation of lignin by 2030 is estimated to be 225 Mt [2]. However, only a small amount of residual lignin extracted annually is recovered (0.075 Mt) from biorefinery and paper production processes (soda lignin) [3], both industries being the largest producers of high lignin streams [4]. For example, the production of pulp through the kraft process generates 1.7–1.8 tons of black liquor per ton of cellulose pulp obtained on a dry basis. Thus, 170 Mt of this liquid, rich in polymers such as lignin, is extracted worldwide in the pulp and paper industry, with an estimated 70 Mt of lignin from the pulping process [5]. On the other hand, the cellulosic ethanol industry extracts 0.5–1.5 kg of lignin per liter of ethanol obtained [6].

Although most of the lignin is incinerated (approximately 98%, [4]), in recent years the retrieved lignin is attracting increasing interest. Thus, assuming a compound annual growth rate of 2.6% from 2023 to 2030, the projected global revenue of the lignin industry for 2030 is estimated to be USD 1217.7 billion [7].

Industries such as pulp and paper, forestry and agro-food are the largest users of lignocellulosic biomasses [8] and, hence, producers of large quantities of plant-based agroforestry industrial biological wastes (lignocellulosic agro-industrial wastes, LAIWs). The recovery of biopolymers, such as lignin, from these waste materials in a biorefinery offers a possibility for the integral valorization of lignocellulosic biomasses.

Recently, lignin from residual lignocellulose materials has aroused enormous interest for being an abundant, low-cost and biodegradable material with high thermal stability and high carbon content [9], which justifies a large number of applications in different areas at an industrial level (obtaining high-value chemical products, energy, intermediary compounds in chemical synthesis, etc.). For this reason, the recovery of lignin from LAIWs is the main subject of study of numerous research projects, taking into account the basic principles of sustainability and the circular economy, since both the integral use of waste and the minimization of non-renewable raw material consumption are two key aspects, within the framework of an environmental perspective, waste management and the promotion of the bioeconomy. However, on the one hand, the great structural complexity of this biopolymer and, on the other hand, the greater abundance of cellulose in lignocellulosic biomasses compared to lignin justifies the fact that experimental studies have so far focused mainly on the use of cellulose, while lignin appears as an undervalued by-product and not as a co-product in these processes.

Therefore, it is of great interest to expand knowledge based on promising routes for obtaining and recovering the lignin fraction of LAIWs, biomasses that are currently of little practical use and that could offer a potential green pathway for the use of lignocellulosic materials, in line with current environmental care policies. In this sense, although many research articles undertake the study of processes and treatments to exploit lignocellulosic wastes, there is a great dispersion of information on aspects related to the valorization of the lignin fraction of these waste biomasses, so this review aims to give a concrete and structured vision, focused on the study of this biopolymer present in agro-industrial wastes (LAIWs), from its obtaining and characterization to its potential applications, offering a complete and updated summary of the advances in research produced over the last fifteen years.

Finally, it should be noted that the general consideration of LAIW as waste does not mean that there are currently no successful alternatives for its valorization but that, in most cases, additional options should be proposed for the by-products of the agro-food industry instead of incineration or disposition on fields, as is the case of 12.1% of biomass wastes [10], because they are considered low-value materials [11].

## 2. Lignin in Lignocellulosic Agro-Industrial Wastes

**Figure 1:**
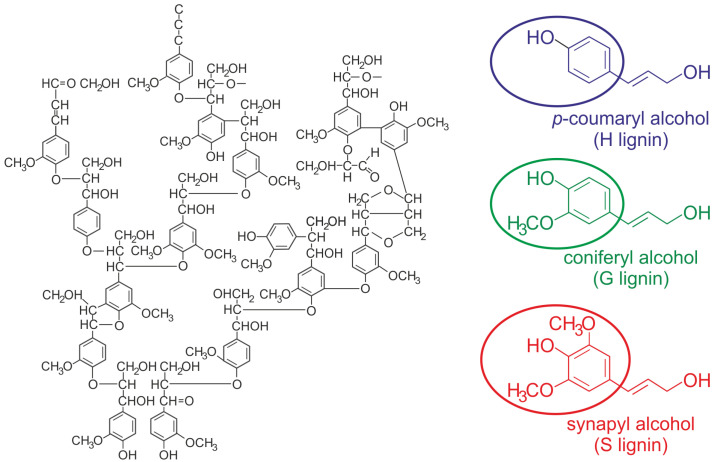
Basic structure of lignin in lignocellulosic biomass (**left**). Different phenylpropane units of lignin (**right**): p-coumaryl alcohol (p-hydroxyphenyl unit, blue circle), coniferyl alcohol (guaiacyl unit, green circle) and sinapyl alcohol (syringyl unit, red circle).

Lignin is, even today, a polyphenolic biopolymer that is not fully understood as its structural complexity is associated with the type of biomass considered, Figure 1. In its structure, different types of functional groups such as carboxyl (-COOH), methoxyl (-${\mathrm{OCH}}_{3}$) and carbonyl (C=O) can be distinguished, as well as different monomers such as p-hydroxyphenyl (H), with little or no presence in soft and hardwoods but this is not the case in grasses, as well as guaiacyl (G) and syringyl (S) units in different proportions, depending on the type of lignocellulosic biomass considered (hardwoods, softwoods, or herbaceous plants) [2]. According to the information from several references, there are differences in the percentages in which these units appear, so hardwoods contain significant amounts of aromatic S units (50–75% *w*/*w*) and G (25–50% *w*/*w*) and a very minor fraction of H (<8% *w*/*w*), softwoods are very rich in G units (>90%), while grass can contain all three types of units: S (20–55% *w*/*w*), G (30–80% *w*/*w*) and H (33% *w*/*w*) [12,13,14].

Some authors point out that the presence of S-units in lignin has a positive effect on increasing the yield of pulp in the paper-making process [15]. In addition, several investigations have generally shown the benefit of a high S/G ratio in lignin depolymerization processes [16], by increasing the amount of labile ether bonds [17], as well as in enzymatic saccharification processes by decreasing the recalcitrance of lignocellulosic biomass [18,19]. In particular, S-lignin features a higher level of labile β-O-4 linkages which are readily cleavable during pretreatment, as well as S-lignin where a relatively higher occurrence of β-$\beta^{\prime }$ bonds leads to a lower molecular weight which could facilitate lignin migration and removal [16]. Vermass et al. [20] observed the maximum expansion and solvation of lignin for solvents with a polarity close to that of dimethyl sulfoxide. In addition, they stated that increasing the syringyl content reduces the solubility of high syringyl lignin polymers. However, other authors concluded that an increase in the S/G lignin subunit ratio did not have a significant positive effect on the enzymatic hydrolysis of lignocellulosic materials [21].

On the other hand, the existence of a higher H-unit content in lignin could imply a decrease in the molecular weight of this polymer as a higher presence of the H-monomer in lignin could facilitate the formation of dilignol compounds, which do not favor chain elongation, leading to polymers with a lower molecular weight [22].

The variety in the composition and structure of lignin for different types of lignocellulosic biomasses determines its heterogeneity, as an identifying characteristic. However, in general and despite their non-homogeneity, two general types of bonds can be distinguished in their structure:

1. Ether bond (C-O-C) which can be originated either between two carbon atoms of different benzene rings or between the propane chain of a phenylpropane (elementary unit often referred to as monolignol) and a carbon of a different benzene ring. One type of bond related to the last situation is the β-O-4 alkyl–aryl ether, considered the most common interaction in lignin (≃40–60% of connections [4,8]), but weaker than other bonds in lignin. A not-too-high dissociation energy for β-O-4 alkyl–aryl ether (289 kJ/mol) [23] allows the biopolymer to break at that position during depolymerization processes. However, α-aryl ether bonds are hydrolyzed around 100 times faster (due to a smaller bond dissociation energy, 215 KJ/mol) than β-aryl ether bonds.
2. Carbon–carbon (C-C) bonds between aliphatic chains of different benzenes or between a benzene and another aliphatic chain. This type of link is more difficult to break which accentuates the recalcitrant nature of lignocellulose biomass, with 5–5′ and β-$5^{\prime }$ bonds representing a high frequency of occurrence, 10–20% and 10–12% of connections, respectively [4].

In general, the term LAIWs referred to in this review encompasses solid lignocellulosic wastes from agricultural, forestry and some food-related industries such as fruit, beer, oil or cereal-processing industries [24], as well as mainly liquid waste streams from the pulping-paper and cellulosic ethanol production industries. After crop harvesting, a multitude of agricultural wastes are produced such as cobs, husks, leaves, pods, prunings, roots, seeds, shells, stalks, stems, stover, straw, stubbles, etc., while in the food industry new wastes appear such as pomace, skin, bran, peel, seed pulp, bagasse, or brewer’s spent grain [24]. These wastes are known as renewable, abundant and highly available raw materials, with potential great applications and growing use at an industrial level.

The lignin composition of forest residual biomasses is highly variable, but generally ranges from 25 to 35% for conifers (gymnosperms such as pine), 18–25% for broadleaves (dicotyledonous angiosperms such as *Acacia*, *Sweetgum* and *Eucalyptus*) and 15–25% for herbaceous biomass (monocotyledonous angiosperms) [2,9]. Furthermore, while hardwoods preferentially contain S- and G-type monolignols, conifers are preferentially rich in S-type subunits [25,26].

Concerning lignocellulosic agro-wastes, produced from the agricultural sector, they mostly come from cereal, grain and legume crops, and represent a global annual production of approximately 5 billion tonnes [27], with Spain being the largest producer of agricultural waste globally [10]. In the case of industrial lignocellulose residues, they come from forestry, agro-food industries and, mainly, from paper pulp factories, especially those using kraft processes [8]; also the fuel industry based on bioethanol production should be taken into account. It is important to mention that agro-food industrial wastes often consist of a mixture of different wastes with different composition and characteristics [10], so their use as feedstock could be complicated, but an effort should be made to use them as their lignin content, in some cases, is considerable [28].

Since LAIW composition varies even for the same type of residual lignocellulosic material, Table 1 provides information on the composition (dry basis) of the three main structural fractions for these materials together with production data. In general, the lignin content of some LAIWs is very significant, especially for acai seed, banana peel, hazelnut shell, palm kernel shell and walnut shell. Some authors report, for lignocellulosic biomasses, cellulose contents in the range (38–50%), hemicelluloses (23–32%) and lignin (15–25%) [11,29,30]. These three biopolymers in LAIWs are tightly bounded by both covalent and hydrogen bonds, forming a highly compact structure that is difficult to break, if treatment conditions are not suitable. The total amount of lignin available annually is very high (5–36 × $10^{8}$ tons) [11], and it could be estimated for each type of waste material taking into account both its average lignin composition and the biomass annual production, according to the data provided in Table 1. Furthermore, it has also been reported that cellulose, hemicellulose and lignin together account for approximately 90% of the dry weight of lignocellulosic materials, while the remaining part comprises proteins, ash and extractives [31]. These extractives include compounds such as flavonoids, lipids, resins, steroids, tannins, terpenes, terpenoids and phenolic compounds [32]. Generally, extractives are more prevalent in leaves, roots and barks, while ash content varies, being typically less than 1% in shells and up to 25% for straws and husks [31].

## 3. Global Process for Lignin Extraction

**Figure 2:**
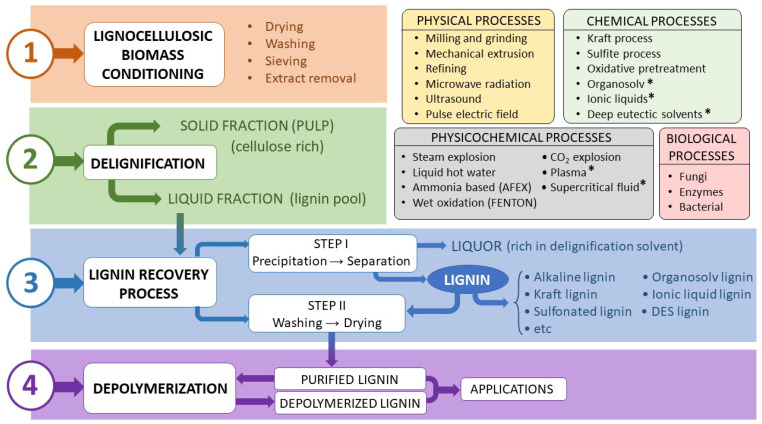
Treatments applied to LAIWs to obtain depolymerized lignin (^*^ green processes).

Extracting lignin from LAIWs involves treatment processes of the original biomass which, applied in a stepwise manner, achieve four main objectives: initial conditioning of the material, lignin solubilization, lignin recovery and depolymerization processes (Figure 2). These depolymerization processes, applied to extracted lignin, attempt to break down its complex polymeric structure into smaller, more useful molecules. Depending on the extraction methods and treatment conditions, lignin has different properties that will condition future applications. In general, more severe conditions will lead to lignin fragments with a different molecular weight.

### 3.1. Methods for Lignin Extraction

Treatments applied for LAIW breakage are usually classified as mechanical, chemical or biological, although in many cases a combination of them can be performed. The final objective will be to depolymerize the material, facilitating the recovery of the three structural fractions of LAIWs, separately. In this sense, specific information for the three main groups of treatments will be discussed.

#### 3.1.1. Physical Treatments

The main objective of physical treatments is to modify some mechanical properties of LAIWs, in order to make them more suitable for subsequent processes. They are targeted at material conditioning by reducing the particle size and crystallinity of the biomass, increasing its specific surface area and porosity, and also altering the biomass structure and polymerization degree of the cellulose [162,163]. In general, they have the disadvantage of high energy expenditure, which increases costs. In research studies (years between 2019 and 2022) based on the valorization of this type of biomass, milling is the most employed process (68%), followed by grinding (16%), refining (9%), ultrasonication (4%) and mechanical extrusion (3%) [164].

For LAIW milling and grinding processes, the equipment used, such as knife, hammer or ball mills, needs the biomass to be either low humidity or dry to prevent the sieve from clogging, as the material is introduced between the knife or hammer rotor and a static sieve, with a specific mesh size, that determines the final particle size and biomass homogenization [165]. Refining is carried out by means of a disk refiner composed of two disks, one static and the other dynamic (connected to a rotor), which moves the biomass towards the periphery. The inside of the disks has special internal tools for size reduction (depending on the disk spacing and speed of rotation); the material is reduced in size as it travels from the center to the periphery. Screw extrusion is a treatment applied to wet biomass in such a way that the material is moved from the inlet to the outlet by the spiral movement of a screw, reducing the size of the material by tearing and friction with the walls and, in turn, decreasing the moisture content by raising the temperature of the biomass. This operation can alter the structure and increase the specific surface area of the raw material, favoring subsequent fractionation treatments such as hydrolytic processes [166]. The ultrasonication process causes a decrease in the local pressure in the liquid, where the biomass is immersed, by the action of ultrasound radiation. If the liquid pressure decreases, to values lower than those of the vapor pressure, then bubbles are generated increasing in size by absorbing ultrasound radiation and, finally, producing a shearing effect in LAIWs when bubbles explode. This breaking effect will be accentuated by the action of free radicals released when the aqueous medium decomposes. This process could delignify LAIWs and remove the hemicellulosic fraction from LAIWs, increasing the cellulose yield of the resulting biosolid [166]. Finally, microwave radiation can interact with LAIWs in such a way that the electromagnetic field generated by the radiation aligns with both induced dipoles produced in waste biomass and ions present in the medium. This fact causes a dissipation of energy that increases the temperature of the biomass [167], leading to the alteration of the cellulose and lignin structure.

In addition to the preliminary mechanical operations mentioned above, other LAIW conditioning operations can be considered, such as washing to remove impurities and pigmented matter, sieving to select biomass fractions with a specific particle size, drying to reduce moisture content, removal of extractives, etc., in order to precondition waste biomass before it is subjected to the specific delignification process.

#### 3.1.2. Chemical Treatments

The purpose of these treatments is to promote the maximum recovery of high-purity lignin from LAIWs, it being necessary to select the most suitable methods and solvents for extraction, as well as to optimize the main experimental variables (solvent concentration, temperature, reaction time, etc.) [168]. By means of the so-called combined severity factor, it is possible to measure the joint effect of these variables, usually defined in acid treatments, Equation (1).

(1) $$\mathrm{Combined}\;\mathrm{Severity}\;\mathrm{Factor}\;\left(CSF\right)\;=\;\log\;\left(\int_{o}^{t}e^{\frac{T\left(t\right)-\;T_{ref}}{w}}\;dt\right)\;-\;pH$$

where *t* is the reaction time, *T* is the temperature at which the delignification process takes place, $T_{ref}$ is the reference temperature (100 °C), *w* is an empirical parameter related to the activation energy (with usual value of 14.75 for LAIWs), and pH is the initial pH value of the solution [10] or final pH value [169], depending on the referenced research study. Other authors [170] consider, for the calculation of the combined severity factor, the term pOH instead of pH for alkaline treatments.

Chemical treatments usually include hydrolysis, oxidation and condensation reactions. These reactions aim to break down native lignin, generating smaller lignin fragments (technical lignin), and use them as raw materials for multiple applications, thereby achieving the delignification of the material. Efficient single-stage treatments for chemical delignification applied on LAIWs would generate a liquid fraction (lignin pool) rich in dissolved lignin, together with oligosaccharides, simple sugars, inorganic matter and degradation products, but also a residual solid (pulp), consisting mainly of cellulose although residual amounts of lignin and hemicellulose could also appear. These chemical treatments are considered as “chemical pretreatments” in biorefinery research, as they are used as a preliminary step to the main treatment (enzymatic hydrolysis). Although great research effort has been made to study the effect of pretreatments on LAIWs, there is still a need to deepen our knowledge in this regard. Recently, there has been more intensive research using different chemical agents and technologies, whether through the use of single or combined processes, which will be discussed in the course of this review.

The main strategies for lignin extraction from LAIWs are divided into two main categories in the literature, depending on their consideration from an environmental point of view: one group consists of traditional (conventional) methods, some of them with industrial use [171], that extract lignins with fewer applications due to the presence of sulfur in their composition. The presence of organosulfur compounds in lignin could greatly limit its use in industrial processes, e.g., in adsorption processes, when catalysts are required, due to the catalysts inactivation [4]. Apart from that, bad odors in products can also be produced. Another group of treatments is the so-called environmentally friendly techniques, emerging technologies leading to the extraction of sulfur-free lignins. Concerning conventional or traditional methods, with greater environmental repercussions, some relevant information is worth bearing in mind related to the main technologies involving lignin extraction.

- Kraft process: Known as sulfate pulping, this is a chemical treatment used in 85% of cellulose pulp production processes from lignocellulosic materials [170,172,173], with an annual lignin extraction volume in the range of 50–70 million tonnes [174,175]. This process aims to extract lignin by using an aqueous solution (white liquid) of sodium hydroxide (NaOH) and sodium sulfide (${\mathrm{Na}}_{2}$S), in the presence of sodium sulfate, although this is not the active form that reacts, and temperatures between 140 and 180 °C [176,177]. The sodium sulfate is used to obtain ${\mathrm{Na}}_{2}$S. The initial pH of the process is close to 13, although this depends on the initial concentration of the alkaline solution. At this pH, more than half of the ${\mathrm{Na}}_{2}$S is in the form of sulfide ions, which are hydrolyzed during the treatment to generate ${\mathrm{HS}}^{-}$ and ${\mathrm{OH}}^{-}$ ions to a pH of 10–12 at the end of the kraft process. Both ions (${\mathrm{HS}}^{-}$ and ${\mathrm{OH}}^{-}$) will attack the biomass, causing the recovery of lignin in the resulting black liquid [178,179], by lowering the pH with ${\mathrm{CO}}_{2}$ or sulfuric acid, among other techniques, until its precipitation. The lignins extracted will have a molecular weight between 1000 and 15,000 g ${\mathrm{mol}}^{-1}$, and will be soluble in both alkalis and organic solvents [177]. Other authors [180] state that the lignin content in the black liquor from the kraft pulp manufacturing process is in the range of 30–45% wt.
  Another more sustainable alternative, using oxygen in some stages of the kraft process, has been proposed in order to reduce the demand for other chemical agents such as sodium sulfide, bleaching agents or chlorine-derived compounds, and also recover some chemicals from effluents generated in the process, as this stream would be free of corrosive chloride ions [177].
  Some authors establish the composition of the white liquid by means of the concept “sulfidity” [25]. This technical term is an important parameter in the pulp and paper industry and affects the efficiency of the cooking process and the quality of the final product. It is calculated as the ratio between the amount of sodium sulfide and the amount of active alkali (NaOH + ${\mathrm{Na}}_{2}$S) in percentage, Equation (2), and it is usually expressed on a ${\mathrm{Na}}_{2}$O basis. A value of 20% for this parameter is recommended in the case of hardwoods (*Eucalyptus*), although this optimum value is strongly dependent on the type of biomass, alkali charge, treatment temperature and final product characteristics [178].
  (2) $$\mathrm{sulfidity}\;\left(\%\right)\;=\;\frac{g\;{\mathrm{Na}}_{2}S\;\mathrm{as}\;g\;{\mathrm{Na}}_{2}O}{g\;{\mathrm{Na}}_{2}S\;\mathrm{as}\;g\;{\mathrm{Na}}_{2}O\;+\;g\;\mathrm{NaOH}\;\mathrm{as}\;g\;{\mathrm{Na}}_{2}O}\;100$$
  Although the kraft process has very important advantages in generating a highly purified cellulose pulp (depending on the operating variables), offering easy reagent recovery, it also has drawbacks to overcome such as low yields in the biomass conversion into cellulose, high production costs and corrosiveness in digesters [178]. Apart from that, the lignin generated has 1–3% sulfur due to the incorporation of aliphatic thiol groups in its structure [181], causing a characteristic odor in this type of lignin [176] and environmental pollution; some studies indicate that 70–75% of the hydroxyl groups may be sulfonated [181]. Furthermore, kraft lignin attainment involves severe conditions producing fragmented but also repolymerizated (condensated) lignins, with higher amounts of recalcitrant carbon–carbon bonds and hydroxyl groups, and less ether linkages, making it difficult to break them up, and even less selective [170]. Finally, the precipitated cellulosic pulp could exhibit surface adherences of condensed lignin, decreasing the purity of the pulp obtained. Therefore, approximately 95% of all kraft lignin is currently burned as a low value fuel [170]. In other cases, the extracted kraft lignin can have annexed hemicellulose-related residues, leading to lignins with amphiphilic behavior like surfactants [177].
- Sulfite process: Together with hydrolysis processes, this accounts for 5% of commercial technical lignin [172], representing 1 million tonnes per year [175]. LAIWs can react with a mixture of alkaline earth metal sulfites at 120–180 °C and high pressure for 3–7 h [174]. Sulfite can be formed by reaction between ${\mathrm{SO}}_{2}$ and calcium carbonate or magnesium oxide at a pH of 1 to 5 [177]. This treatment allows the extraction of lignins of an amphiphilic nature, containing both hydrophilic groups such as sulfonates, ${{\mathrm{HSO}}_{3}}^{-}$ (highly polar), or hydroxyl groups, ${\mathrm{OH}}^{-}$ with hydrogen bonding capability, but also hydrophobic groups, encompassing aromatic rings and alkyl groups that have relatively non-polar character. The balance between hydrophilic and hydrophobic groups in sulfite lignin determines its overall solubility and compatibility with other materials. It is considered that the high presence of sulfonate groups makes sulfite lignin more hydrophilic than other types of lignin, such as kraft lignin, which has fewer ionic groups. Therefore, sulfite lignins can form polyelectrolytes because they contain ionizable functional groups (sulfonic, carboxyl and phenolic hydroxyls) when dissolved in water, giving rise to a molecule with multiple negative electrical charges. The electrostatic repulsion between the negative charges of the sulfonate groups causes the lignin chain to expand, occupying a larger volume in the solution. This also enhances the solubility of lignin in water through ion–dipole interactions. Subsequent hydrolysis of the formed sulfonates allows lignin to be solubilized in water. These lignosulfonates will have a high molecular weight (1000–50,000 g ${\mathrm{mol}}^{-1}$), sulfur content of 2.1–9.4%, mainly in the benzylic position, and high ash percentage (4.0–8.0%) [181,182]. On the other hand, polyelectrolytes could interact with cations to give rise to chemical complexes that modify the solution properties.
- Klason treatment: This is a chemical process that involves a first stage of treatment with concentrated sulfuric acid (72%) for 1 h at 30 °C, and a second hydrolysis stage of the residual solid with dilute sulfuric acid (4%) for 1 h at 121 °C [183]. The main drawback of the process is that the extracted lignin is highly altered, so it is only used as a quantitative method at an experimental level.
- Acid treatments: These processes, historically established and appealing due to their cost-effective and high efficiency, have limited utility for biomass delignification. This limitation arises because these pretreatments are optimized for hemicellulose recovery rather than maximizing solubilized lignin extraction. Several industrial companies carry out acid treatments of waste biomass to generate second generation biofuels, although this technology is not yet consolidated, which justifies that research is still ongoing [184]. Many studies use traditional mineral diluted acids such as hydrochloric, nitric, phosphoric and especially sulfuric acid (which is the most employed acid) considering acid concentrations in the range 0.2–8% (*w*/*w*), liquid:solid ratios from 5:1 to 20:1 (*v*/*w*), temperature range from 80 °C to 210 °C, and reaction times from 5 to 300 min [184,185] in order to preferentially solubilize hemicellulose, amorphous cellulose or soluble lignin, but leading to cellulignin solids in which lignin is still present to a considerable extent. Therefore, hydrolytic treatments with diluted inorganic acids could be of interest only in the case of LAIWs with a low lignin content; using diluted inorganic acids, only delignification percentages of 5–10% can be achieved [184]. Studies using more concentrated acids in the hydrolytic process tend to use lower temperatures (<60 °C), higher solid:liquid ratios from 1:2.5 to 1:10 (*w*/*v*) and acid concentration of more than 10% (*w*/*v*) [12,185,186]. Some authors mention in their studies acid concentrations as high as 65–86% (*w*/*v*) sulfuric acid, 41% (*w*/*v*) hydrochloric acid or 85% (*w*/*w*) phosphoric acid [187], and times in the range 2 to 10 h under atmospheric pressure [12]. Problems of equipment corrosivity, partial cellulose solubilization (especially amorphous fraction), mandatory need to recover the acid and production of undesirable compounds (e.g., inhibitors), in residual liquid streams, contribute to the environment pollution and cost increase. For these reasons, other environmentally friendly alternatives, employing weak organic acids such as formic, maleic, acetic, oxalic, citric, propionic or lactic acids, have been proposed. Some of these acids (formic, acetic, propionic or lactic) are considered “organosolv” because of their low toxicity, low cost and high recoverability. Specifically, Singh et al. [188] employed wheat straw and bagasse by applying a sequential treatment with concentrated sulfuric acid and then dilute acid, achieving lignin recovery percentages of 21.5% for one of the bagasse types. Table 2 presents the experimental conditions and delignification percentages of the most recent studies.

**Table 2:** Specific experimental conditions for acid delignification treatments using LAIWs.

LAIWs	Experimental Conditions	Delignification, %	Ref.
Sugarcane straw	Acetic acid 7.51 g LAIW-6.7 mL acid + 20 mg	91.0	[189]
Coffee husk	NaCl four times, 60 min, 80 °C	80.0	
Corn cob	p-Toluenesulfonic acid 70%, 70 °C, 30 min	82.4	[52]
Corn stover	Acetic acid 2%, 12 ± 2 h, 15 psi, 121 °C, 90 min 1 g LAIW-15 mL acid	75.7	[190]
Empty palm fruit bunch	${\mathrm{HNO}}_{3}$ 2% *w*/*v*, 150 °C, 30 min	65.7	[191]
Rice husk	$H_{2}{\mathrm{SO}}_{4}$ 2% (*v*/*v*), 120 °C, 60 min 1 g LAIW-10 g acid	18.8	[192]
Rice straw	$H_{2}{\mathrm{SO}}_{4}$ 0.75% (*v*/*v*) + Boric acid 1% (*w*/*v*) + glycerol 0.5% (*v*/*v*) 1 g LAIW-5 mL acid, 150 °C, 20 min	44.0	[193]
	Acetic acid 99%, 80 °C, 24 h 1 g LAIW-10 mL acid	4.7	[29]
	p-Toluene sulfonic acid 60% (*w*/*w*), 80 °C, 45 min	52.4	[194]
Sugarcane bagasse	Formic acid 80.0%, 130 °C, 90 min 1 g LAIW-10 mL acid	90.6	[195]
	$H_{2}{\mathrm{SO}}_{4}$ 1.5% (*v*/*v*), 121 °C, 20 min 1 g LAIW-10 mL acid	33.1	[196]
Soybean hull	Citric acid 4.22%, 120 °C, 105 min 1 g LAIW-15 mL acid	30.4	[149]
Wheat straw	p-Toluenesulfonic acid 15%, 25%, 35% (*w*/*w*) 90 °C, 120 min	24, 48, 66	[197]
	Maleic acid 60% (*w*/*w*) 110 °C, 60 min	66.5	[198]
	Sulfuric acid 1.5% (*w*/*w*) 121 °C, 15 lb/inch^2^, 75 min	74.0	[199]

**Figure 3:**
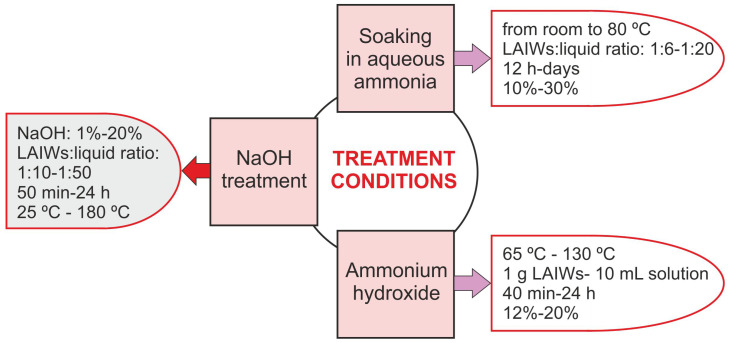
Conditions of the main alkaline treatments for LAIWs.

Currently, research has been intensified to find new technologies for using LAIWs to extract lignin, avoiding sulfur compounds that can cause environmental and corrosion problems. Research in this field is continuously developing, as the choice of the most appropriate method will depend on both the type of biomass and the desired properties of the lignin. Some of these technologies carry the label “green”, especially referring to processes using hot water, organic solvents, supercritical fluids, non-thermal plasma, ionic liquids, deep eutectic solvents, microwave-assisted extraction and biological treatments [4,81,200]. The main methods to extract sulfur-free lignin include the following technologies:

- Alkaline (AK) treatments: One of the best known hydrolytic processes is the so-called “soda pulping”, the preferred pulping process for agro-waste fractionation [174]. This chemical method uses NaOH solutions (10–16%) at high pressures and temperatures (140–170 °C) to produce the alkaline hydrolysis of LAIWs and extract the lignin (soda lignin) although anthraquinone is sometimes added in order to stabilize the hydrocelluloses [174,177]. It is traditionally applied to non-wood matrices like straw or bagasse [11] and allows obtaining 15% of the lignin marketed [172], with an annual extraction of 6000 tonnes [175]. In this case, the extracted lignin (soda lignin) is lighter in color, more soluble in water and has lower sulfur content compared with kraft lignin. Furthermore, it has a low molecular weight (generally 1000–3000 g ${\mathrm{mol}}^{-1}$) and methane emissions are produced [201]. The application of soda extraction processes leads to efficient depolymerization of the material, extracting alkaline lignin (AL) with very high purity [202] and zero sulfur content [181]. Furthermore, soda lignins contain more hydroxyl groups and relatively more carbon–carbon bonds compared to native lignin [177]. The values of the combined severity factor for soda pulping are usually in the range of 0.3–4.5 for this type of process [170], so that an increase in the degree of delignification from 25.3% to 95.4% has been evidenced when the severity varies from 1 to 5, respectively. However, from combined severity factor values above 3, lignin starts to be degraded, undergoing changes in its structure as condensation reactions take place [170]. In this sense, poplar wood hydrolysis solid was subjected to alkaline lignin extraction (NaOH, 1 M at 75 °C, 3 h) recovering 48% lignin [203]. Ammonium hydroxide, anhydrous ammonia, sodium carbonate and sodium sulfite have also proven to exhibit LAIWs delignification, Table 3. Figure 3 resumes the conditions of the main alkaline treatments.

**Table 3:** Experimental conditions for alkaline delignification treatments using LAIWs.

LAIWs	Experimental Conditions	Delignification, %	Ref.
African oil palm fiber Coffee pulp waste Sugarcane bagasse	Ca(OH)_2_ 0.1% (*w*/*w*) 40 °C, 1 g LAIW-28 mL solution	31.3 35.0 32.8	[204]
Banana stem	KOH 1–5% (*w*/*w*), 90 °C, 25 min 1 g LAIW-10 mL solution	43.0	[205]
Sugarcane bagasse Coconut husk Rice straw Corn stover	NaOH 3% 1:12 (*w*/*v*) 121 °C, 15 psi	31.3 38.5 19.4 15.8	[202]
Chestnut shell	NaOH or KOH: 1–5% (*w*/*w*)	15.5–51.1	[206]
Corncob	${\mathrm{KMnO}}_{4}$ 0.5–4% (*w*/*v*) 30–70 °C, 1–8 h 1 g LAIW-10 to 30 g solution	46.8	[205]
Corn stalk	NaOH 1.5% (*w*/*v*), 121 °C, 30 min 1 g LAIW-10 g solution	42.2	[207]
Corn stover	${\mathrm{NH}}_{4}$OH 12% (*w*/*w*) 130 °C, 40 min 1 g LAIW-10 mL AK solution	75.1	[208]
	${\mathrm{NH}}_{3}$ 29.5% room temperature, 60 days	73.5	[209]
	${\mathrm{NH}}_{3}$ 15.0% 60 °C, 12 h	62.0	[209]
	${\mathrm{NH}}_{3}$ 15.0% 90 °C, 1 g LAIW-12.5 mL solution	68.9	[210]
	${\mathrm{NH}}_{3}$ 15.0% (*w*/*w*) 60 °C, 12 h 1 g LAIW-10 mL AK solution	70.0	[211]
Groundnut shell	NaOH 1.75 N 80 °C, 6 h, 400 rpm 0.8 g LAIW-100 mL AK solution	41.8	[212]
Miscanthus giganteus	NaOH 0.16–1.0 mol/L 100–180 °C, 60–360 min, 360 rpm	25.3–95.4	[170]
Olive pomace	NaOH 8% (*w*/*v*), 50 °C, 24 h 1 g LAIW-59 mL AK solution	68.0	[213]
Pistachio shell	NaOH 1.75 N 80 °C, 6 h, 400 rpm 0.8 g LAIW-100 mL AK solution	38.0	[212]
Rice hull	${\mathrm{NH}}_{3}$ 20%, 100 °C, 1 g LAIW-10 mL	62.0	[214]
Rice straw	${\mathrm{Na}}_{2}{\mathrm{CO}}_{3}$ 8% 140 °C, 1 g LAIW-6 g solution	41.8	[208]
	${\mathrm{NH}}_{3}$: 5% 121 °C, 60 min, 15 psi 1 g LAIW-10 mL solution	78.4	[215]
	${\mathrm{Na}}_{2}{\mathrm{CO}}_{3}$ 2–10% (*w*/*v*) 80 °C, 180 min, 15 psi 1 g LAIW-10 mL solution	28.3–60.5	[216]
Sugarcane bagasse	NaOH 5, 10, 15% KOH: 5, 10, 15% 98 °C, 90 min 1 g LAIW-10 mL AK solution	5.0–17.4 9.7–18.3	[217]
	Urea/KOH (1:1 ratio) 3–12% −20 °C, 1–12 h 1 g LAIW-(20–50) g AK solution	41.5	[218]
	${\mathrm{Na}}_{2}{\mathrm{CO}}_{3}$ 1.5% (*w*/*w*) NaOH 1.5% (*w*/*w*) 121 °C, 20 min 1 g LAIW-10 mL solution	35.3 38.3	[196]
	NaOH 6% (*w*/*w*), 90 °C, 60 min 1 g LAIW-15 mL solution	81.0	[219]
	${\mathrm{NH}}_{4}$OH 20% (*v*/*v*) 70 °C, 24 h	41.5	[220]
Sugarcane straw	NaOH 1% (*w*/*v*) ${\mathrm{Na}}_{2}{\mathrm{SO}}_{3}$ 10% (*w*/*v*) ${\mathrm{NH}}_{4}$OH 10% (*w*/*v*) 120 °C, 60 min, 1 g LAIW-10 g AK solution	28.5 25.1 46.3	[221]
Sweet sorghum residue	NaOH 2% 121 °C, 50 min, 15 psi 1 g LAIW-10 mL AK solution	41.8	[222]
Wheat straw	NaOH 0.1 M room temperature, 120 min 1 g LAIW-10 mL solution	21.1	[223]
	${\mathrm{NH}}_{4}$OH 15% (*w*/*w*) 65 °C, 15 h 1 g LAIW-10 mL solution	50.0	[224]
	${\mathrm{NH}}_{3}$ 15% (*w*/*w*) room temperature, 7 days 1 g LAIW-20 mL solution	9.0	[225]

- Hydrotropic extraction: A novel development for the fractionation of LAIWs is the use of hydrotropic compounds in aqueous solution, that contain hydrophilic and hydrophobic groups (with very short chains), such as (sodium or potassium) alkyl benzene sulfonates, xylene sulfonates, toluene sulfonates, salicylates and urea. These compounds have the ability to increase the solubility of lignins, that are normally insoluble in water, during the initial treatment phase. Subsequently, the lignin may be precipitated by diluting the hydrotrope solution with water [226] so, in many experimental studies, quite acceptable delignification percentages of LAIWs have been obtained, Table 4.

**Table 4:** Experimental conditions for hydrotropic extraction treatments using LAIWs.

LAIWs	Hydrotropic Solvent	Experimental Conditions	Delignification, %	Ref.
Rice straw	Sodium cumene sulfonate 20%	1 g LAIW-20 g acid, 121 °C, 1 h, 50%	50.0	[227]
	Sodium xylene sulfonate 20%		34.0	
Sugarcane bagasse	Sodium xylene sulfonate 40%	170 °C, 1 h	6.8	[228]
	Sodium xylene sulfonate 30%	1 g LAIW-2 g hydrotrope 115 °C	85.0	[229]

- Organosolv treatment: Process to extract technical lignin from LAIWs, using several organic solvents (30–100% *w*/*w*), solid–liquid ratios in the range 1:2 and 1:15, although 1:10 balance is the most common, temperatures in the range 100–250 °C (temperatures > 180 °C are required for higher delignification) and residence times from 30 to 60 min [177,230,231]. This treatment is implemented on a pilot scale for raw biomass with an annual organosolv lignin extraction of 1000 tonnes [175]. One such method is the “Alcell” process, which uses ethanol: water mixtures (1:1) at 180–210 °C and 2–3.5 MPa to solubilize lignin from lignocellulosic biomasses [232]; the “Alcell” process has evolved into the “Lignol” process whose main difference is the addition of an inorganic acid (e.g., sulfuric acid) to maintain the pH in the range 2–3.7 [233].
  Other organosolv pretreatment with the addition of catalysts is becoming the focus of much research for LAIWs [230]. Although ethanol is the most common solvent involved due to its advantageous properties (low cost and toxicity, high quality of the recovered lignin and high recyclability [231,234]), other organic solvents are involved, such as methanol, 1-butanol, isobutanol, 2-methyltetrahydrofuran (MeTHF), tetrahydrofuran (THF), glycerol, ethylene glycol, cyrene, triethylene glycol (TEG), polyethylene glycol (PEG), acetone, formic acid, acetic acid, dimethyl isosorbide, ethyl lactate and γ-valerolactone, being used for LAIWs depolymerization and subsequent lignin recovery. Although there is research based on dioxane treatments, its use in industrial processes has been severely restricted due to the risks it poses to human health and the environment. Organosolv treatment achieves, in many cases, high lignin removal rates (>70%) with low loss of cellulose (<2%) [235], but research needs to address their main disadvantages: high material cost, solvent volatility and solvent recovery cost [236]. Table 5 shows organosolv experimental conditions and grades of delignification for different LAIWs.

**Table 5:** Experimental conditions for organosolv delignification using LAIWs.

LAIWs	Organosolv Solvent	Experimental Conditions	Delignification, %	Ref.
Bamboo culms	Formic acid:acetic acid:water 30:50:20 *v*/*v*/*v*	1 g LAIW-20 mL OS 60 °C, 1 h + 107 °C, 3 h	31.5	[237]
Corn stalks	Formic acid:acetic acid:water 60:30:10 *v*/*v*/*v*	1 g LAIW-5 g OS 90 °C, 180 min	18.6	[238]
Corn	Methanol 0–2.5% *w*/*v*	1 g LAIW-2 mL OS	55.0	[239]
stover	(alkali catalyzed)	80 °C, 60 min	70.7	[240]
Corn straw	Cyrene 50% *w*/*w*	120 °C, 60 min	78.0	[231]
Cotton stalks	Formic acid 100% $H_{2}O_{2}$ 2% *w*/*w*	1 g LAIW-10 g OS 80 °C, 60 min	81.0	[207]
Rice straw	Glycerol 90% *w*/*w* (salt catalyzed)	1 g LAIW-20 mL OS 150 °C, 20 min	71.9	[241]
Sugarcane bagasse	Ethylene glycol 90% *v*/*v* (acid catalyzed)	1 g LAIW-10 mL OS 150 °C, 60 min	67.1	[242]
	Ethylene glycol 90% *v*/*v* (acid catalyzed)	1 g LAIW-20 mL OS 120 °C, 60 min, 1% HCl	61.2	[243]
	Ethanol 60% *v*/*v* (acid catalyzed)	1 g LAIW-10 mL OS 170 °C, 15 min, 25 g ${\mathrm{CO}}_{2}$	86.0	[244]
	Ethanol 60% *v*/*v* (alkali catalyzed)	1 g LAIW-10 mL OS 180 °C, 30 min, 8% NaOH	82.6	[245]
	TEG 5% *w*/*v* (alkali catalyzed)	1 g LAIW-20 mL OS 90 °C, 120 min, 1% NaOH	80.0	[246]
Wheat straw	Water–ethanol 3:7 to 1:9 *v*/*v*	1 g LAIW-25 mL OS 90 °C, 120 min	50.0	[247]
	Glycerol 70%	1 g LAIW-10 g OS 220 °C, 3 h	65.0	[248]
  Recently, the role of certain organic compounds to prevent lignin repolymerization processes has been discovered. Thus, compounds such as 2-naphthol and dimethyl phloroglucinol have been used to stimulate delignification processes, protecting lignin from condensation reactions [249].
  These compounds can yield lignin practically free of sulfur (0–0.3% [181]), molecular weight of 500 to 5000 g ${\mathrm{mol}}^{-1}$ and also having high purity and reactivity, preserving almost completely the chemical structure of the natural lignin [177] without condensation. Furthermore, organosolv lignin has a high hydrophobic character, so it is practically insoluble in water favoring lignin precipitation processes for its recovery. Research is currently prioritizing the application of this method due to the low environmental impact of the process and the high purity of the lignin recovered, although further study is needed to improve the economic aspects of the process, for example, towards the recovery of the solvents used. In this sense, only solvents with low boiling points are easy to recycle by evaporation, but they are volatile and highly flammable with requirements of expensive high-pressure equipment. On the other hand, solvents with high boiling points present recovery problems; impurities can appear frequently [231].
- Ionosolv treatment (IL): Chemical treatment that intends to use ionic liquids, salts with low melting point (usually <100 °C) [4], to recover lignin (and hemicellulose) from LAIWs, leaving the cellulose unaltered. Other advantageous properties of ionic solvents, apart from being in a liquid state at a low temperature, encompass their non-volatility and non-flammability, high conductivity and thermal stability [250], but some drawbacks are also presented concerning the high cost for the production of these solvents [251]. Although some ionic solvents used in the past were labeled as toxic and corrosive (contain anions such as ${\mathrm{Cl}}^{-}$, ${\mathrm{Br}}^{-}$, $I^{-}$, [${\mathrm{BF}}_{4}{]}^{-}$ and [${\mathrm{PF}}_{6}{]}^{-}$), there is now a shift towards more environmentally friendly liquids. According to some studies, the constituent cations of the salts belong to different groups (quaternary ammonium or phosphine salts, imidazole and pyridine), while the most advisable anions are [${\mathrm{CH}}_{3}{\mathrm{COO]}}^{-}$ (acetate), [${\mathrm{NH}}_{2}{\mathrm{CH}}_{2}{\mathrm{COO]}}^{-}$ (glycinate), [$C_{6}H_{5}O_{7}{]}^{3-}$ (citrate), [${\mathrm{HPO}}_{4}{]}^{2-}$ (hydrogen phosphate), [$H_{2}{\mathrm{PO}}_{4}{]}^{-}$ (dihydrogen phosphate), [${\mathrm{CH}}_{3}C_{6}H_{4}{\mathrm{SO}}_{3}{]}^{-}$ (tosylate), [${\mathrm{CH}}_{3}{\mathrm{SO}}_{3}{]}^{-}$ (mesylate), [${\mathrm{CH}}_{3}$CH(OH)${\mathrm{COO]}}^{-}$ (lactate), etc. [251]. When treatments are carried out at high temperatures, phosphonium-based ionic solvents are preferred to imidazolium- and ammonium-based ionic solvents, as the stability of phosphonium salts is higher.
- Deep eutectic solvent (DES) treatment: This is considered an environmentally friendly chemical alternative for lignin extraction as solvents are required have low toxicity, high recyclability and biodegradability; other properties are non-flammability, being 20% cheaper than ionic solvents, high chemical and thermal stability, low vapor pressure, low volatility and biocompatibility [252,253,254]. This process generally consists of a binary system based on two organic components (solids under normal conditions): one of them is a hydrogen bond donor (HBD) and the other a hydrogen bond acceptor (HBA). Both compounds result in a mixture with a melting point much lower than the one for each compound separately [255], due to charge delocalization via hydrogen bonds between molecules of both organic compounds [256]. The main groups of HBA are quaternary ammonium, phosphonium or sulfonium halide salts [257], choline chloride (ChCl) being the most common natural deep eutectic solvent because of the low toxicity of cholinium, although mixtures consisting of choline chloride:glycerol and betaine: amino acid are also frequently reported. Concerning HBDs, the most relevant chemicals are amide, carboxylic acid [257] and polyols [258], but other solvents may also be used. In summary, Table 6 shows various HBAs or HBDs reported in recent studies with DESs. The most commonly used DESs for biomass processing are usually based on the following types of mixtures: quaternary ammonium salt with hydrated metal halide, due to their resistance to air and moisture, and lower melting point compared to non-hydrated forms, as well as quaternary ammonium salts and carboxylic acids for their low cost and easy preparation. Additionally, inorganic transition metals and some HBDs such as urea can also be used. Recently, incipient research is underway considering only non-ionic molecules with ambiguous HBD/HBA roles in DESs [259], as is the case of the combination of N-methylacetamide and a long-chain carboxylic acid (lauric acid) [223]. Microwave radiation-assisted DES treatments have sometimes been used, so that solvents with high electrical conductivity and high dielectric constant favored collisions with lignocellulosic biomass, promoting delignification processes [259].

**Table 6:** Main hydrogen bond acceptors and donors in deep eutectic solvent technology.

Hydrogen bond acceptors (HBAs)	Choline chloride (ChCl), N,N-diethyl-2-hydroxy-N-(2-hydroxyethyl)ethan-1-aminium chloride, Ammonium chloride, Tetra-hydroxymethyl phosphonium chloride (TPC), Tetramethyl-ammonium chloride (TMAC), Tetraethyl ammonium chloride (TAC), Tetrabutylammonium chloride, Tetrabutylammonium hydroxide (TAH), Methyltriphenylphosphonium bromide, Triethyl benzyl ammonium chloride (TEBAC), N-benzyl-2-hydroxy-N,N-dimethylethanamine, Tetraethylammonium chloride, Benzyltriethylammonium chloride (BTEAC), 2-acetate-N,N,N-trimethylethanaminium chloride, Glyoxylic acid (GA), Betaine (Be), Hydrated metal halide: AlCl_3_·6H_2_O; FeCl_3_·6H_2_O; CrCl_3_·6H_2_O, FeCl_2_·4H_2_O	[253,256,258,259,260,261,262,263]
Hydrogen bond donors (HBDs)	Urea, Formamide, Thiourea ethanolamine (EA), Diethanolamine (DEA), Methyldiethanolamine, 3-aminopropanol (AP), Isopropanolamine (IPA), Triethanolamine (TEA), N-methyldiethanolamine (NMDEA), 1,3-diamino-2-propanol (DAP), 2-amino-1,3-propanediol (ADP), 4-hydroxy benzyl alcohol, Ethylene glycol (EG), Propylene glycol 1.4-butenediol, Acetamide (AC), Benzamide, Glycerol, Imidazole, Boric acid (BA), Monocarboxylic acids: glycolic, lactic (LA), levulinic, acetic (AA), formic (FA), propionic, butyric, octanoic, decanoic, Di or tricarboxylic acids: malonic, oxalic (OA), succinic, malic (MA), maleic, glutaric and citric (CA) acids, Glucose, Fructose, Sucrose, Xylitol, Salicylic acid, Gallic acid, p-coumaric acid, Vanillin, p-hydroxybenzoic acid, p-hydroxybenzaldehyde, p-hydroxybenzyl alcohol, Catechol, Resorcinol, Glycerol (Gly)	
Both HBDs and HBAs	Menthol (Men) 2,6-dimethoxyphenol (Dmp)	[99]
  DESs have a high viscosity, due to the existence of hydrogen bonding, electrostatic interactions and van der Waal forces between HBAs and HBDs. Some studies have linked a higher viscosity for those DESs with a higher number of hydroxyl groups, in the chemical composition of HBDs, due to the formation of a higher number of hydrogen bonds [259]. Nevertheless, viscosity can be lowered by both increasing the temperature or dilution with water to break the existing bonds, originating HBD–water interactions, although dilutions above 30% are not advisable [257]. Density is also an important factor for DES mobility and activity: the more hydroxyl groups and the shorter chain length of the HBDs, the higher density of the DESs [264]. In addition, the chemical nature of the HBDs could also condition the effectiveness of the lignin fractionation process. Effectiveness in DESs can be improved if some remarks are considered such as shortening alkyl chains in carboxylic acids, using monocarboxylic acid-based HBDs instead of di- or tri-carboxylic acids, increasing the presence of hydroxyl groups in a-hydroxyl acid-based DES, increasing the amount of amine/amide groups in HBDs and enhancing HBD:HBA molar ratio in DES [259]. In general, the molar ratio between HBDs and HBAs will affect the physicochemical properties (density, viscosity, polarity, melting point, etc.) of the generated mixture [265].
  Recent research has shown the suitability of incorporating co-solvents into the initial eutectic mixtures to increase the efficiency of biomass fractionation. In this sense, the incorporation of substances such as ${\mathrm{AlCl}}_{3}\cdot 6H_{2}$O or small amounts of water to DES systems would favor the fractionation and cleavage of lignin bonds, as a large amount of acid protons could be generated. Apart from that, the addition of water would decrease the viscosity and increase the diffusivity and interactions of DESs in the biomass [259]. Other advances in the field of fractionation with DESs are based on the use of simultaneous technologies, in particular the use of DESs combined with physical treatments such as microwave radiation and ultrasound techniques, promoting both the breaking of chemical bonds and the appearance of cavities or micropores, that favor the action of eutectic solvents.
  Lignins from DES treatment are characterized by a large number of phenolic hydroxyl groups and small molecular size [266]. Although the effectiveness of the use of DESs has been demonstrated, one of the main concerns related to eutectic solvents is that it results in more condensated lignin extraction. Although condensated lignins imply positive aspects for both the manufacture of composite materials and activated carbon production (increased surface area, more functional groups and less rigid structure), they also have serious drawbacks when soluble lignin is required, also hindering enzymatic hydrolysis as undesirable lignin could be deposited on the cellulose surface, hindering the cellulose activity [253]. Several studies have shown that deep eutectic solvents used for lignocellulosic material delignification have resulted in lignins with a large number of hydroxyl groups and high antioxidant activity, therefore promoting their reactivity and use [254]. To minimize the undesired effect of lignin condensation, recent research points to the use of ternary mixtures, e.g., by adding polyol-based solvents, such as ethylene glycol, to certain binary mixtures (choline chloride–oxalic acid). These polyols would react with benzylic intermediates in a β-position with respect to the β-O-4 ether bonds, inhibiting lignin condensation [267]. The precise mechanisms involving DES biomass delignification are still under investigation but the high capacity for DESs to form hydrogen bonds, interacting with lignin functional groups (hydroxyl, phenolic, methoxyl, etc.) against groups present in cellulose and hemicellulose could explain LAIW depolymerization, interacting and leading selectively to the preferential dissolution of lignin [268]. Table 7 summarizes some of the most recent research with DESs for different LAIWs. Although dependent on the type of DES used and the extraction conditions, the lignins extracted will, in general, have greater thermal stability, due to a lower presence of labile functional groups in their structure, with higher molecular weight and more conserved structure, as they are extracted with milder solvents, compared to lignins extracted by traditional basic or acidic methods.
  On the other hand, since around 2020, much research work has focused specifically on the use of lignin extraction methods adapted to preserve the structural characteristics of the lignin in the original material, so that it undergoes as few modifications as possible during the polymer extraction process. In this way, the field of application of the lignins obtained could be extended by applying specific depolymerization processes according to the applications to be given to the lignins obtained.

**Table 7:** Experimental conditions using deep eutectic solvent (DES) technologies for LAIW delignification.

LAIWs	DES	Experimental Conditions	Delignification, %	Ref.
Boehmeria nivea stalks	Be:GA 1:6 molar ratio	1 g LAIW-10 g DES 130 °C, 2 h	81.9	[263]
Brewer’s spent grains	ChCl-LA/ChCl-Gly 1:10; 1:2 molar ratio	1 g LAIW-20 g DES 60–80 °C, 24 h	34.5–39.3	[262]
Camellia oleifera shell	ChCl-EA/ChCl-AP/ChCl-IPA 1:6 molar ratio	1 g LAIW-20 mL DES 90 °C, 1–12 h	59.9	[261]
Corncob	ChCl-Gly/ChCl-urea/ChCl- Imidazole 1:2; 1:2; 3:7 molar ratio	1 g LAIW-16 g DES 115 °C, 15 h	88.0	[269]
	Be-Lysine/Be-Arginine/ Be-Histidine 1:1 molar ratio	1 g LAIW-10 to 25 g DES 60 °C, 5 h	24.4–57.0	[270]
Corn stover	ChCl-LA/TMAC-LA 1:2 molar ratio	1 g LAIW-10 g DES 130 °C, 2 h	62.6	[271]
Corn straw	ChCl-LA/Be-LA 1:2 molar ratio	1 g LAIW-20 mL DES 120 °C, 6 h	32.3–89.7	[272]
Industrial xylose residues	Guanidinium hydrochloride-LA 2:1 to 1:2 molar ratio	1 g LAIW-20 mL DES 120 °C, 2 h	57.5–73.5	[273]
Oil palm empty fruit brunch	ChCl-different carboxylic acids	1 g LAIW-10 g DES 120 °C, 8 h	>60.0	[274]
	ChCl-LA 1:2 molar ratio	1 g LAIW-10 mL DES 100–120 °C, 10-30 min	9.1–57.1	[275]
Reed straw	BTEAC-FA 1:2 to 1:6 molar ratio	1 g LAIW-10 g DES 130 °C, 3 h	78.1	[276]
	Be:LA:H_2_O 1:5:5 molar ratio	1 g LAIW-20 g DES 80 °C, 24 h	52.0	[277]
Rice straw	MA-proline/ChCl-OA/ChCl-urea 1:3; 2:1; 1:2 molar ratio	1 g LAIW-10 g DES 120 °C, 4–12 h	35.0–75.0	[278]
	ChCl-glycerol 1:3 to 1:9 molar ratio	1 g LAIW-19 g DES 80–150 °C, 3–24 h	53.3–74.2	[279]
Sugarcane bagasse	ChCl-LA/ChCl-CA/ChCl-AA 1:4 to 4:1 molar ratio	1 g LAIW-15 mL DES 130 °C, 90 min	2.7–54.5	[280]
	ChCl-OA/ChCl-trifluoracetic acid 1:2 to 1:1.5 molar ratio	1 g LAIW-19 g DES	51.0–56.0	[281]
	ChCl-LA/Be:LA 10–5% *w*/*w*	1 g LAIW-9 g DES 140 °C, 2 h	16.6–39.4	[282]
Wheat straw	ChCl-CA-EG; ChCl-MA-EG; ChCl-FA-EG; ChCl-AA-EG; ChCl-BA-EG; ChCl-LA-EG	1 g LAIW-20 g DES 80–140 °C, 3–24 h	35.7–92.4	[253]
	TEBAC-Gly-Al chloride 1:2:0.05 molar ratio	10 g LAIW-3% DES 100 °C, 16 h	1.2–7.8	[283]
	Gly-K_2_CO_3_ 1:5 molar ratio	1 g LAIW-10 g DES 100–140 °C, 1 h	70.5–83.0	[284]

- Oxidative treatments: These promote delignification processes using chemical oxidants, Table 8, such as alkaline hydrogen peroxide ($H_{2}O_{2}$), wet oxidation, organic peracid pretreatments, Fenton oxidation and ozone technology [234]. As this process takes place under conditions of medium severity, the formation of inhibitory compounds is more unlikely than other promising technologies with options to be implemented on an industrial scale (such as steam explosion).
  Hydrogen peroxide is considered by many authors to be an effective treatment that promotes sustainability [285]. This oxidative compound, in strongly alkaline media, generates hydroxyl (·OH) and superoxide radicals (${\cdot O_{2}}^{-}$), with a strong oxidizing character, encouraging lignin depolymerization. While medium-severity conditions in oxidative treatments with $H_{2}O_{2}$ allow attacking mainly the hydrocarbon chains of lignin, more severe treatments would oxidize aromatic rings. It has been noted that, at pH higher than 10, the effectiveness of the hydrogen peroxide is high but no delignification was achieved below this value [286].
  These hydroxyl radicals are also produced by the Fenton process based on the oxidation, in a more efficient way, of LAIWs using ${\mathrm{Fe}}^{2+}$ and hydrogen peroxide under acidic conditions, to avoid the loss of ${\mathrm{Fe}}^{2+}$ reagent by the formation of complexes of this cation or the formation of Fe(OH)_3_, in the case of basic conditions [287]. Treatment by wet oxidation implies using oxygen (or air) and water at 120–238 °C and 3–35 air bars with lower production of inhibitors but higher production of collateral products, compared to other techniques (e.g., steam explosion) [234].

**Table 8:** Experimental conditions using oxidative technologies for LAIW delignification.

LAIWs	Oxidative Treatment	Experimental Conditions	Delignification, %	Ref.
Brewer spent grains	Hydrogen peroxide (alkaline conditions)	1–8% $H_{2}O_{2}$, 20 °C, 0–12 h, pH = 11.5 1 g LAIW-12.5–50 g oxidant	17–36	[288]
Chinese hickory shell	Hydrogen peroxide (alkaline conditions)	0–3 mL $H_{2}O_{2}$ (30%), 20 °C, 300 rpm, 3 h 1 g LAIW-15 mL oxidant	50.0	[289]
Oil palm empty fruit bunch	Fenton	200 mM $H_{2}O_{2}$, 24 h, room temperature 1 g LAIW-50 mL oxidant	71.2	[287]
Rice husk	Hydrogen peroxide (alkaline conditions)	1% $H_{2}O_{2}$, 5.3% NaOH, 20 °C 0–12 h, 150 rpm	59.6	[290]
Sisal waste	Ultraviolet-catalyzed hydrogen peroxide (alkaline conditions)	0.05–0.4 g $H_{2}O_{2}$/g LAIW, pH = 10 1 g LAIW-20 mL oxidant	76.2	[291]
Toonna sinensis branches	Hydrogen peroxide + acetic acid (100 mM as catalyzer)	12% $H_{2}O_{2}$, 37.6% acetic acid, 170 °C, 90 min	77.0	[292]
Wheat straw	Hydrogen peroxide (alkaline conditions)	4% $H_{2}O_{2}$, 110 °C, 2 h, pH = 11.5, 400 rpm 1 g LAIW-40 mL oxidant	70.7	[293]

#### 3.1.3. Physicochemical Treatments

- Liquid hot water (also known as autohydrolysis [294]) is a hydrothermal method focused on the solubilization mainly of hemicellulose (with the generation of oligosaccharides and monosaccharides), although the solubilization of smaller amounts of lignin can also be achieved; in particular, delignification percentages of 20–30% can be reached [295]. Pressurized water is used to carry out the hydrolysis of LAIWs involving an auto-catalyzed process by both hydronium ions, generated by the ionization of water, and the action of acetic acid formed from the acetyl groups, released from the hemicelluloses. Additionally, the way in which lignin depolymerization takes place is strongly dependent on the experimental conditions (mainly temperature, reaction time and solid:liquid ratio). Although more severe conditions would imply higher degrees of delignification of residual biomasses, bioproducts that are difficult to control would also be extracted, so typical conditions (140–240 °C and liquid:solid ratios of 10, [12,294,296]) are geared towards biofuel more than lignin extraction. Frequently, the terms “subcritical water” and “liquid hot water” are often used interchangeably to describe water that is at temperatures above its normal boiling point but below its critical point. From a more general point of view, this treatment consists of using water at temperatures between 100 °C and 374 °C and pressures high enough to keep it in a liquid state (under 1–20 MPa for 10–50 min) [296]. Some researchers have shown that the use of subcritical water as a one-stage method is not effective the for delignification of LAIWs [297] and they are frequently used for both bioactive compounds and sugars production. Nevertheless, delignification data (13.73%) have been obtained using mango seed shell (180 °C, 15 min, 2.5 MPa) [298]. More recent studies employ subcritical water in combination with other techniques such as deep eutectic or ionic solvents [299], obtaining significant delignification, especially by the contribution of other solvents apart from water [300], Table 9.
- Steam explosion method: This is a hydrothermal treatment considered as an environmentally friendly technique for four relevant reasons: the main agent used is water vapor, an abundant and renewable resource, high energy efficiency, low use of chemical reagents (diluted acid or alkali) and recovery of almost intact lignin. The disintegration of the biomass occurs by abrupt decompression after reaching high temperatures and pressures, usually in the ranges (160–270 °C) and (10–50 bars), respectively [39,301]. Residence times (2–40 min) and biomass properties (moisture content or particle size) are also determining factors in the depolymerization process of LAIWs [301,302,303]. Considering the reduction in fermentation inhibitors, the combination of a low temperature and longer residence time may be a better choice. In contrast, the option of a short residence time at high catalyst concentrations is less ideal [163].
  Finally, the concentration of the main catalysts ($H_{2}{\mathrm{SO}}_{4}$, ${\mathrm{CO}}_{2}$ and ${\mathrm{SO}}_{2}$) used to increase efficiency is strongly dependent on the type of catalyst, nature of biomass and purpose of the treatments. Most of the research studies, using this hydrothermal treatment, have focused on experimental conditions to enrich the solid resulting from steam explosion treatment in cellulose, for subsequent enzymatic hydrolysis and biofuel obtainment, as well as treatments for xylo-oligosaccharides or anaerobic gas production. In this sense, considering sugarcane bagasse depolymerization by acid catalyzed steam explosion [304] uses phosphoric acid concentrations between 0.05 and 0.4% (*w*/*v*) to disintegrate the structure of sugarcane bagasse. However, when using ${\mathrm{SO}}_{2}$, concentrations in the range of 0.9-3% are used to treat the same material.
  In the latest LAIW delignification studies, the steam explosion technique has been used in combination with other main chemical, physicochemical or biological processes [303], as shown in Table 9, concerning combined biomass treatments, to increase the efficiency of the global lignin removal process.

**Table 9:** Parameters and delignification percentages for different LAIWs using hydrothermal processes.

LAIWs	Hydrothermal Method	Experimental Conditions	Delignification, %	Ref.
Barley straw	Deionized water (subcritical)	60% *v*/*v*, 180 °C, 50 bar 5 mL/min	61.0	[305]
Canola straw	Deionized water (subcritical)	60% *v*/*v*, 180 °C, 50 bar, 5 mL/min	51.0	[305]
Chestnut shell	Liquid hot water	100 °C, 24 h 1 g LAIW-10 mL water	18.8	[306]
Rice straw	Subcritical water	170–200 °C, 4.67 mL/min	85.0	[307]
Sugarcane bagasse	Steam explosion (NaOH)	0.1 M calatyst 180 °C, 5 min, 20 bar	65.0	[308]
	Liquid hot water	180 °C, 20 min, 160 psi 1 g LAIW-9 mL water	12.8	[309]
Sugarcane straw	Steam explosion	Deionized water soaked 2 h at 20 °C 210 °C, 15 min, 20 bar	6.0	[310]
Sugarcane trash	Steam explosion (NaOH)	Double impregnation: 1 g LAIW-10 mL water 1 g-20 mL NaOH 17%, 190 °C, 17 min	70.5	[311]
Wheat bran	Steam explosion	0.05 (*v*/*w*) $H_{2}{\mathrm{SO}}_{4}$ 218 °C, 8 min	25.1	[228]
Wheat straw	Liquid hot water	180 °C, 40 min 1 g LAIW-10 mL water	21.6	[312]
	Liquid hot water	160–200 °C, 30–90 min	10–35	[313]
	Subcritical water	121 °C, 45 min, 15 lb/inch_2_	57.0	[199]
- Supercritical or subcritical fluids: Supercritical fluids (carbon dioxide, ammonia, water, acetone, ethanol, methanol, and hydrocarbons such as propane and butane) are obtained when they are subjected to pressures and temperatures above their critical point, so that there is no transition phase and the liquid and solid phases become indistinguishable. Therefore, these fluids are chemical compounds that have properties of both liquids and gases [314] and advantageous characteristics such as low viscosity, low dielectric constant and high diffusivity [4], improving the solubility of hydrophobic compounds in water and encouraging lignin fractionation [315]. In the case of ${\mathrm{CO}}_{2}$, one of the most commonly used fluids due to its low cost, non-toxicity and a relatively low critical point, temperatures above 31.7 °C and pressures of 7.38 MPa are required [186]; however, water requires conditions above 374 °C and 221 bar [314]. In some cellulose and biofuel research, the delignification conditions must be carefully chosen, so that the yields in carbohydrate fraction are high as well as the quality of the recovered collateral lignin [316].
  Recently, the use of supercritical fluids, especially ${\mathrm{CO}}_{2}$, in combination with other delignification treatments, has been advocated, as it is possible to preserve the structure of the lignin to a greater extent. Some experimental conditions for one-step treatments are shown in Table 10, exceptionally collecting delignification results from research prior to the time interval for which the review has been conducted.

**Table 10:** Parameters and delignification percentages for different LAIWs using critical fluids.

LAIWs	Fluid	Experimental Conditions	Delignification, %	Ref.
Barley straw	Ethanol	60% *v*/*v*, 220 °C, 50 bar, 40 min 5 mL/min	60.0	[52]
	Ethanol	20% *v*/*v*, 180 °C, 50 bar 5 mL/min	54.0	[305]
Canola straw	Ethanol	20% *v*/*v*, 180 °C, 50 bar 5 mL/min	45.0	[305]
Corn stover	${\mathrm{CO}}_{2}$ (supercritical)	ethanol-water 2:1 *v*/*v* 200 °C, 13 MPa, 80 min 1 g LAIW:40 mL	90.0	[317]
Hemp fibers	${\mathrm{CO}}_{2}$ (supercritical)	ethanol-water 2:1 molar ratio 180 °C, 6 MPa, 60 min 1 g LAIW:6 mL	89.6	[99]
Rice husk	${\mathrm{CO}}_{2}$ (supercritical)	ethanol-water, 80 °C, 270 bar, 10 min	34–91	[192]
Sugarcane bagasse	${\mathrm{CO}}_{2}$ (supercritical)	190 °C, 16 MPa, 60 min	88.4	[318]
	${\mathrm{CO}}_{2}$ (supercritical)	60 °C, 200 kg_*f*_/cm^2^	8.1	[319]
- Ammonia Fiber Expansion (AFEX): This treatment of LAIWs, with liquid ammonia at pressures of 0.7–2.7 MPa, temperatures of 60–100 °C and residence times in the range 5–60 min, involves rapid ammonia expansion and release of pressure at the end of the treatment, causing swelling of the biomass, changes in the structure of the lignin, some solubilization of the hemicellulose and partial delignification [12,209,320,321]. When concentrated ammonia is brought into contact with water, ammonium and hydroxide ions appear in solution, through an exothermic reaction that increases the temperature of the reaction medium. The simplicity of ammonia recovery makes this treatment very useful. However, some research reports a lack of effectiveness of the delignification process for some LAIWs such as corn stover [322] or agave bagasse [323]. No additional information on recent delignification data of LAIWs has been found by applying this process apart from those listed in Table 11.
  A modification of the AFEX process is the ammonia recycle percolation (${\mathrm{NH}}_{3}$ in continuous recirculation and recycling in a percolation reactor) [324]. Experimental conditions for this process usually comprise 140–210 °C, residence time 14–90 min, 5–15% (*w*/*w*) of ammonia solution and velocity of 1 cm $\min^{-1}$ [324,325] for woods. Soaking aqueous ammonia is also considered a modification of the AFEX process by using batch reactors, but under milder temperature conditions (25–60 °C) and longer reaction times (10–60 days).

**Table 11:** Experimental conditions and delignification data for different LAIWs using AFEX process.

LAIWs	AFEX Conditions	Delignification, %	Ref.
Corn stover	${\mathrm{NH}}_{3}$ 58.7% *w*/*w* 30 °C, 10 min 0.5 H_2_O_2_ loading (30%)	24.0	[326]
Pine chips	70% moisture 130 °C, 15 min 1 g LAIW: 1 g ${\mathrm{NH}}_{3}$	4.0	[327]
Wheat straw	10 g-7 mL deionized water 130 °C, 15 min 1 g LAIW: 1 g ${\mathrm{NH}}_{3}$	3.8	[328]

- Hydrodynamic cavitation: This treatment allows, using non-rotational systems (orifices, Venturis, vortex diodes, swirling jet), or advanced rotational reactors to provoke liquid contraction, the growth and collapse of vapor bubbles in the solvent in which the residual biomass is found, by a pressure drop below the saturation pressure of the liquid. In this process, pressures of ∼500 bar, temperatures of ∼5000 °C and oxidation processes (by hydroxyl radical formation) are presented [329], causing mechanical and chemical effects on the biomass, and producing its disintegration.
  A hydrodynamic system has been used [330] to assist different alkaline pretreatment processes for sugarcane bagasse, achieving 48.31% delignification using KOH (0.5 mol/L) at 60 °C, 20 min, dropping the pressure from 3 bar to 0.3 bar in the flowing fluid through the orifice plate. The same experimental conditions but using NaOH (0.3 mol/L) produced a delignification of 43.63%. On the other hand, information collected from Iskalieva et al. [331] summarizes experimental conditions for LAIWs treated by this technique, generally in conjunction with other chemical processes, showing percentages of lignin release, solubilization or removal in the range 2.2–78.5%.
- Non-thermal plasma (cold plasma) technique: Although there are also high temperature processes, low-temperature plasma (in the range 25–100 °C) [81] are regarded as being more environmentally friendly, especially more than those using chemical agents, although its novelty justifies the need for further studies to be optimized [332]. Cold plasma processes are generally used in combination with other chemical treatments. In addition, the biomass can undergo mechanical as well as chemical depolymerization, if there is the possibility of free radical formation which can oxidize LAIWs. This technique is characterized by the presence of electrically charged particles, a mixture of electrons, ions, radicals and neutral particles (in varying proportions depending on full or partial ionization) by the action of an electrical discharge with sufficient voltage on a gas (air, oxygen, helium, air–argon, ozone, etc). The interactions produced between the electrons (attaining temperatures of 105–5000 °C) and the gas molecules also generate active free radicals that attack the biomass and lead to its degradation [333]. The main advantage is that, although electrons reach extremely high temperatures, the gas surrounding them is at a relatively low temperature, so that no dissociation of the reaction products is observed and no cooling is necessary. In addition, this technique avoids problems of environmental damage, waste generation and inhibitors production, in contrast to many of the commonly used chemical treatments. Its proven efficiency in lignin extraction, low cost and its non-toxic character make it a promising strategy in lignocellulosic biomass fractionation processes.

#### 3.1.4. Combined or Assisted Methods

The combined (hybrid technologies) or assisted methods are intended to represent improved processes for extracting higher quality lignin, making it a promising strategy in LAIW fractionation technologies. The combination of several methods could overcome the individual limitations of each process and lead to lignins with more specific and selective properties. Table 12 summarizes the main combined delignification treatments recently applied to different lignocellulosic biomasses, also providing information on the experimental conditions of the treatment and the type of feedstock used in the study.

#### 3.1.5. Thermochemical Treatments

Lignin can be obtained by thermochemical treatments of LAIWs, submitted to two main processes: pyrolysis and hydrothermal liquefaction [9]. Although pyrolysis involves the thermal decomposition of biomass in the absence of oxygen, which might suggest a classification as a physical treatment by many authors [346,347], the nature of the chemical transformations that occur during this process could place it firmly in the category of thermochemical treatments. Depending on the experimental conditions, several types of pyrolysis can be distinguish—slow pyrolysis (carbonization) which occurs at <500 °C, heating rates of 0.1–1 °C/s and treatment times (5–30 min), with a higher possibility of generating high levels of charcoal; fast pyrolysis with high heating rate (10–200 °C/s), temperature of 400–800 °C, treatment times less than 2 s and bio-oil yields in the range 50–70%; and flash pyrolysis with higher heating rate ($10^{3}$–$10^{4}$ °C/s), temperatures in the range 800–1000 °C, residence times lower than 0.5 s and bio-oil yields of 75–80% [348,349,350]—although these temperature ranges and heating rates vary, and even overlap, depending on the source consulted [350]. In general, according to Bajwa et al. [174], low temperature and heating rate promote char production, short residence time and high heating rates yield preferably liquid products but high temperature, long residence time and low heating maximize gas production from biomass.

Pyrolysis, therefore, is usually conducted in a non-oxidative atmosphere, high temperatures and very short times, justifying that lignin from pyrolysis is highly deconstructed and appears in the form of undersized chemical compounds in the three main fractions obtained (bio-oil, but also biochar and non-condensable gases) [351]. Concretely, bio-oil (usually produced by fast pyrolysis) from empty fruit bunches consists mainly of carboxylic acids, phenols and ketones, although furans and esters are also present [352]. However, bio-oil could also contain lignin fragments (20% of pyrolytic lignin from woody biomass, apart from compounds consisting of carboxylic, carbonyls and phenolic groups) [9,351], depending on the temperature used, due to the decomposition of hemicellulose that occurs between 250 and 350 °C, cellulose at 325–400 °C and lignin at 300–500 °C [349]. A first water extraction of the bio-oil allows the separation of the water-soluble fraction of pyrolytic lignin from the heavier fraction. This denser fraction can be subjected to molecular distillation to remove the water and then the pyrolysis lignin can be precipitated with methanol [353], but some inconveniences can occur such as the thermal polymerization of phenol, aldehyde and lignin oligomers [9].

An important advance in lignocellulosic biomass pyrolysis processes is the use of zeolites as catalysts, although others such as activated carbon or metal-based catalysts have also been investigated [354]. Specifically, the molecular size of the three-dimensional structure of zeolites can allow the selective diffusion of molecules of a specific size, favoring reactions that can increase, for example, the yield of bio-oil.

The hydrothermal liquefaction of LAIWs would allow lignin to be obtained by treating the biomass with water at temperatures between 250 °C and 374 °C, and pressures of 4–22 MPa [9], even if LAIWs have a high moisture content, obtaining a bio-oil named bio-crude, residual solid (hydrochar), aqueous phase (containing glycolic acid, acetic acid, methanol, ethanol, ketones and cyclopentanones, and inorganic materials) and gases such as ${\mathrm{CO}}_{2}$, ${\mathrm{CH}}_{4}$, $H_{2}$ and CO [355,356]. Other authors extend the temperature and pressure range to 200–550 °C and 5–25 MPa for agricultural and forestry residues [355]. Santos Silva et al. [357] studied this treatment using sugarcane bagasse and straw with the purpose of obtaining the best experimental conditions (ranges of 300–350 °C, 0–30 min, 0–0.5 mol/L^−1^ of catalyst, $K_{2}{\mathrm{CO}}_{3}$) to produce bio-oil, considering 200–300 °C, 3 h and cooper as the catalyst [358]. Although some authors consider hydrothermal liquefaction as a method of lignocellulosic biomass delignification, it has not been possible to find information on lignin losses specifically for LAIWs, in order to be recovered as such, instead of their final degradation products.

#### 3.1.6. Biological Treatments

Biological delignification treatments are processes that use microorganisms, mainly bacteria and fungi, due to the capacity of specific enzymes to break down the lignin present in LAIWs. The specificity of some microorganisms towards lignin, leaving other valuable components of the biomass intact, the low severity of the experimental conditions required and the low environmental impact of these processes are encouraging their use, although there is room for improvement in some aspects related to the speed of the processes and their cost. Enzymatic hydrolysis is usually applied to biomass that has undergone preliminary treatments to improve the digestibility of the enzymes in hydrolytic routes, using post-treated solids, enriched in cellulose, as starting materials. In these hydrolytic processes, both the crystallinity index of the enriched cellulose material and its degree of polymerization are important, as these parameters will determine the efficiency of the process. The lower both the crystallinity index and the degree of polymerization of the cellulose, the easier it is to carry out enzymatic hydrolysis [359].

The enzymes involved in cellulose hydrolysis belong to the family of endo-glucanase, exo-glucanase and β-glucosidase. Each of the enzymes has a specific role; briefly, endoglucanase randomly cleaves internal β-1,4-glycosidic bonds in cellulose chains; exoglucanase acts on the ends of cellulose chains, removing cellobiose; and β-glucosidase hydrolyses cellobiose into two glucose molecules. Depending on the pretreatment method, the lignin-containing subsequent stream may be solid or liquid. If in the liquid fraction, lignin can be recovered by precipitation. Otherwise, if in the solid fraction, after hydrolyzing with cellulolytic enzymes, lignin-enriched solids will be obtained that can be depolymerized for processing into lignin-derived products.

Nowadays, the principles of the circular economy promote the harnessing of the lignin contained in both liquid and solid waste flows. In this respect, sugarcane bagasse is subjected to a sequential process consisting of hydrothermal treatment (190 °C, 10 min and a solid: liquid ratio of 1:10) and subsequent enzymatic hydrolysis, recovering a solid with 47.31% of lignin [360]. In the same way, fungal pretreatment (Mn-assisted) of wheat straw and maize stover achieved delignifications of 34% and 37.9%, respectively [361]. In particular, white-rot fungi possess the ability to degrade lignin thanks to the production of enzymes such as laccase, among others. In this sense, the enzymatic delignification of pineapple leaf waste is carried out by laccase produced from *Lentinus squarrosulus* MR13. In this process, a delignification percentage of 81.12% is achieved for a solid loading of 21.5% (*w*/*v*), pH = 5.1, 44 °C and enzyme concentration of 3500 IU/g. Andlar et al. [362] collect in their study data from previous studies on the biological delignification of different LAIWs, using microorganisms: fungi such as *Pleurotus ostreatus* and *Phanerochaete chrysosporium* used in the solid-state fermentation (15 days) of cotton stalks achieve delignifications of 45% and 35%, respectively. Additionally, previous works collected in Saritha et al. [363], using *Ceriporiopsis subvermispora* on corn stover, show delignification ranges of 31.6–39.2%. Some bacteria have also demonstrated their efficacy in removing lignin from LAIWs, as in the research of Shen et al. [216] in which using a combined treatment of ${\mathrm{Na}}_{2}{\mathrm{CO}}_{3}$ and B8 bacterium leads to delignification data of 22%.

Some processes, such as EnZolv pretreatment, combine physical and biocatalytic methods by first subjecting the LAIWs to a steam treatment to disrupt its complex structure and, secondly, laccase enzymes act in the biomass for lignin degradation. In this sense, Subramaniam et al. [364] study cotton stalk delignification reaching lignin removal of 68.68%.

### 3.2. Lignin Recovery and Depolymerization

When processes for biomass fractionation and delignification are carried out, final streams containing both lignin and materials resulting from cellulose and hemicellulose depolymerization are produced. Therefore, separation techniques (e.g., filtration) [365], after treatments are often used to differentiate delignified LAIW fiber (solid) and lignin-rich black liquor (lignin pool). The global process for lignin recovery from liquid solutions after delignification treatments occurs by different separation stages, applied sequentially: precipitation, separation, washing and drying of the final lignin. One of the most industrially used techniques for the recovery of lignin persisting in pools of lignin is the LignoBoost process [366], initially applied for the separation of kraft lignin. This process consists of two sequential stages: firstly, the aim is to recover the lignin by precipitation, decreasing the pH of the black liquid, with ${\mathrm{CO}}_{2}$, using this compound so as not to alter the equilibrium between ${\mathrm{Na}}^{+}$ and $S^{=}$ ions in the precipitation process. During this stage, the phenolic groups in the lignin are protonated as the pH drops and polymer precipitation is favored. After filtration, the recovered crude lignin is further washed in a second stage with an acidified solution [25]. Another recent process for the isolation of kraft lignin from black liquor is the “LignoForce” process. For separation, the liquid is subjected to an oxidation process, then acidification with ${\mathrm{CO}}_{2}$ to cause lignin precipitation, and filtration to separate the black liquor and recover a solid cake to be washed [367].

In general, the most common methods for the precipitation of lignins from the liquids resulting from the LAIW fractionation process involve different possibilities: on the one hand, acidification processes of lignins, when they are present in alkaline media, using acids such as sulfuric acid to lower the pH from 13–13.5 to 5–7.5. Concerning kraft lignin, it is hydrophobic in character and precipitation can occur by acidification and precipitation [201] although its dark color and the presence of sulfur in its composition may limit certain applications. On the other hand, carbon dioxide acidification is another option, which can lead to cost reductions if the ${\mathrm{CO}}_{2}$ involved in the main process (kraft process, for example) can be recycled. However, lignin precipitation yields with ${\mathrm{CO}}_{2}$ could be lower as the final pH achieved is not lower than 8, so the lignin can only be partially recovered. Other feasible techniques for lignin recovery would be electrodialysis, which uses membranes selective to certain ions (cations or anions), or ultra- and nanofiltration [174]. Table 13 summarizes the methods to obtain separated biopolymer from the pool of lignin.

On the other hand, the application of lignin depolymerization processes is a crucial step for the efficient valorization of this biopolymer [266]. The idea is to advance the challenge of converting polymers into more basic constituents, avoiding the defunctionalization of these lignin derivatives, in order to reconstruct from them new basic polymers or obtain chemicals with adequate quality and characteristics. The depolymerization efficiency of lignin into smaller molecules (monomers or oligomers) will depend on the quantity of β-O-4 bonds, since breaking these bonds requires less energy than C-C bonds, so that it can be accomplished under mild experimental conditions through various methods such as DES depolymerization, pyrolysis, alkaline methods, hydrogenolysis, electrochemical depolymerization, oxidation, hydrolysis, gasification, biological processes, etc. [266,348]. The conditions used to carry out lignin depolymerization processes as well as the separation and purification of the bioproducts are very important challenges in recent research.

In this sense, deep eutectic solvents, apart from dissolving the lignin and contributing to the fractionation of the biomass, allow the depolymerization of the lignin by breaking some ester and aryl ether bonds found in its structure. Therefore, as an example, lignin depolymerization using amino acid-based deep eutectic solvents as hydrogen bond acceptors and glycerol as the hydrogen bond donor (1:3 M ratio) fosters the cleavage of both ether and carbon–carbon linkages [368]. The advantage for these processes is that the lignin obtained is very similar to the original lignin [369]. Concerning organosolv depolymerization, two-step acid–alkali lignin from empty palm fruit bunches is deconstructed with mixtures of water: ethanol (1:1) at 180 °C, 7 MPa and 60 min, rending 37% of monomers [191]. Oganosolv lignin from bagasse can also be depolymerized using methyl isobutyl ketone to obtain high-value-added chemicals [370]. Other treatments based on ionic liquids can also conduct lignin depolymerization, obtaining different aromatic compounds [371]. Polyphenols are also produced using 2-hydroxy ethylammonium (24 h, 110 °C and ratio of 1 g lignin:50 mL solution) from organosolv lignin [372]. Ionic compounds as depolymerizing agents have drawbacks since these solvents interact with the lignin and they have high cost and low recovery, which limits their use [369].

**Table 13:** Experimental conditions for lignin recovery in the pool after delignification.

STEP I: Precipitation → Separation		STEP II: Washing → Drying		LAIWs	Ref.
Precip./Extraction	Separation	Washing	Drying		
POOL FROM ALKALINE PROCESS					
$H_{2}{\mathrm{SO}}_{4}$ 20% *w*/*w* final pH = 2	Centrifugation 4000 rpm 15 min	Hot water	60 °C, 5 days	Bagasse Coconut husk Rice straw Corn stover	[202]
$H_{2}{\mathrm{SO}}_{4}$ 24% *w*/*w* final pH = 4 + enzymatic hydrolysis	Centrifugation 3000 rpm 60 min	5× with buffer to pH = 4	60 °C	Miscanthus giganteus	[170]
$H_{2}{\mathrm{SO}}_{4}$ final pH = 2	Centrifugation 12,000 rpm 15 °C, 20 min	until pH = 7		Sugarcane bagasse	[217]
$H_{2}{\mathrm{SO}}_{4}$, latic and formic acid 4 M pH = 4	Centrifugation	until pH = 3		Sugarcane bagasse	[219]
HCl 12% 70 °C, pH = 2–3	Centrifugation 9000 rpm	until pH = 7	60 °C	Green sandalwood	[365]
$H_{2}{\mathrm{SO}}_{4}$ 30%	Vacuum filtration		Spray drying	Sugarcane bagasse	[373]
POOL FROM KRAFT PULPING					
LignoBoost using ${\mathrm{CO}}_{2}$ pH = 9.5	Centrifugation	HCl pH = 2.5 + acetic acid pH = 3.5	Freeze-dried	Pine Acacia Eucalyptus	[25]
HCl 1 M pH = 2 Ethanol pH = 7, 8 h	Vacuum filtering		Dried 45 °C, 24 h	A. mangium	[374]
POOL FROM DEEP EUTECTIC SOLVENT TREATMENT					
Acetone:water 1:1 Evaporation 60 °C + freezing −80 °C	Rotavapor 60 °C, 30 min		Freeze-dried	Reed	[277]
Deionized water precipitation	Centrifugation $10^{3}$ rpm, 5 min	Ethanol:water 1:9 *v*/*v*	Freeze-dried	Wheat straw	[253]
Cooling + Deionized water precipitation	Centrifugation and decanting		Lyophilization	Camellia oleifera shell	[261]
Ethanol:water 1:1 *v*/*v*		Distilled water to pH = 7	Freeze-dried	Rice straw	[375]
Ethanol:water 1:2 *v*/*v*	Centrifugation	Distilled water 2 times	Oven dried 40 °C	Oil palm fruit bunch	[274]
Ethanol 3 times	Rotavapor 40 °C deionized water filtration	Deionized water 2 times	Oven dried 40 °C	Reed straw	[276]
Ethanol:water 9:1 *v*/*v*	Rotavapor	Ultrapure water 3 times	45 °C, 24 h	Brewer’s spent grains	[262]
NaOH 0.1 M room temperature	Centrifugation + decantation	Distilled water 3 times	Lyophilization	Corncob	[270]
Ethanol:water 1:9 *v*/*v*	Centrifugation	Ethanol:water 1:9 *v*/*v*		Corn straw	[272]
Distilled water	Centrifugation		Freeze-dried 48 h	Ind. xylose residues	[273]
Water	Centrifugation	Cold water	49 °C, 48 h	Oil cane bagasse	[282]
Deionized water	Vacuum filtered	Deionized water, pH = 7	70 °C	Oil palm fruit bunch	[275]
POOL FROM COMBINED TREATMENT					
Acetone 96%	Rotavapor 35 °C		40 °C	Defatted grape seed	[376]
$H_{2}{\mathrm{SO}}_{4}$ 20%	Centrifugation 3500 rpm	Water to pH = 2	50 °C, 24 h	Coconut husk	[335]
HCl pH = 2	Centrifugation 8500 rpm	Distilled water, 60 °C	60 °C	Rice straw	[343]
Ethanol or acetone used in previous organosolv treatment	Filtration		Vacuum 80 °C 4 h	Apricot kernel shells	[334]
POOL FROM ORGANOSOLV TREATMENT					
Ethanol	Centrifugation 1200 rpm		Freeze-dried	Corn stover	[337]
Pure water	Centrifugation	Deionized water	Freeze-dried 50 °C, 48 h	Corn stover	[377]
Distilled water	Filtration		60 °C, 24 h	Sugarcane bagasse	[244]
Water	Centrifugation		Freeze drying	Bamboo culms	[237]
Acidic deionized water pH = 3–4	Centrifugation 4000 rpm		45 °C	Wheat straw	[247]
Ethyl acetate	Filtration + rotavapor		Vacuum dried 60 °C	Corn stalks	[238]
$H_{2}{\mathrm{SO}}_{4}$ 95% pH = 1.5–2.0	Centrifugation 3220× *g*	Acidified water pH = 2		Persimmon tree pruning waste	[378]
POOL FROM $H_{2}O_{2}$ TREATMENT					
HCl 6 M	Centrifugation		Freeze-dried	Chinese hickory shell	[289]
POOL FROM ACID/ENZYMATIC TREATMENT					
Deionized water pH = 2	Centrifugation 4000 rpm		Lyophilization	Corn stover	[379]
$H_{2}{\mathrm{SO}}_{4}$ 2.5 M 70 °C, 1 h	Filtration	Water wash	80 °C	Poplar wood hydrolysis solid	[203]
POOL FROM SUPERCRITICAL/SUBCRITICAL TREATMENT					
HCl pH = 2	Vacuum filtration	Deionized water	Freeze-dried	Hemp fibers	[99]

Catalytic oxidation involves breaking the chemical bonds of lignin by the addition of oxygen, with the aid of a catalyst in case of homogeneous or heterogeneous catalysis. The cleavage is usually carried out with nitrobenzene, hydrogen peroxide, air, oxygen and some oxides of transition metal (${\mathrm{Fe}}^{2+}$, ${\mathrm{Mn}}^{3+}$, ${\mathrm{Co}}^{2+}$, ${\mathrm{Cu}}^{+}$, etc.) implying a crucial positive aspect related to the preservation of the aromatic ring structure, rendering aromatic polyfunctional monomers such as vanilaldehyde, syringaldehyde and p-hydroxybenzaldehyde on the one side and p-hydroxybenzoic, syringic and vanillic acids on the other hand [380]. Although operating conditions are variable, oxidation of biomass with oxygen or air (2–14 bar) at temperatures in the range (120–190 °C) tends to produce primarily phenolic monomers. In turn, these compounds can undergo more severe oxidation reactions to generate organic acids (formic, acetic, malonic, etc.) using strong oxidizing agents such as $H_{2}O_{2}$ at temperatures between 60 and 225 °C. This type of depolymerization process takes place mainly in alkaline media (NaOH or KOH) although sulfuric, nitric or acetic acid apart from organic solvents (methanol) can be employed [381]. In this line, recent studies employed soda lignin extracted from wheat straw to be subjected to nitrobenzene oxidation for vanillin production [382]. Additionally, organosolv and alkaline lignin, extracted from sugarcane bagasse, has also been considered for the production of vanillin and syringyl aldehyde by heterogeneous catalytic oxidation using $O_{2}$ as oxidant and Pd/γ-${\mathrm{Al}}_{2}O_{3}$ [383]. Additionally, ammonia-extracted lignin from corn stover was alkali-oxided using $O_{2}$ and NaOH (at 100–160 °C, 0.5–1.5 h and 0–2 MPa) to aromatics (vanillin, p-coumaric acid, etc.) and organic acids (formic, oxalic, acetic, etc.) efficiently [384]. Furthermore, lignin was depolymerized by hydrothermal $H_{2}O_{2}$ oxidation using CuO/Fe_2_(${\mathrm{SO}}_{4}$)_3_/NaOH as catalysts in order to produce monophenolic compounds (17.92%) [385]. Finally, lignin can also be oxidized enzymatically since enzymes such as manganese peroxidase and laccase can oxidize phenolic compounds in lignin, while non-phenolic compounds are oxidized by lignin peroxidase and versatile peroxidase [386,387].

Alkaline- and acid-catalyzed depolymerization of organosolv lignin has proved to be an effective treatment to also produce bio-oil and phenolic compounds, such as catechol and phenol [388]. These processes are carried out using strong bases such as sodium or potassium hydroxide or complex bases (NaOH and ${\mathrm{Na}}_{2}{\mathrm{SO}}_{3}$), which are capable of breaking β-O-4 bonds, although the main drawback is the repolymerization reactions which generate a large amount of solids that hinder the depolymerization process. NaOH/urea aqueous solution has also been employed to depolymerize alkali lignin at a low temperature to generate phenol–formaldehyde resins [389]. Apart from that, gas, bio-oil, solid residue and aromatic monomer yields were explored from the depolymerization of kraft and concentrated sulfuric acid hydrolysis lignins, treated with different bases (${\mathrm{Na}}_{2}{\mathrm{CO}}_{3}$, KOH and NaOH) considering different solvents (supercritical methanol and subcritical water) [390]. In line with this, alkaline supercritical water (386 °C and 260 bar) also yielded monoaromatics from lignin recovered according to sequential treatments involving subcritical water extraction followed by an enzymatic hydrolysis step [391].

Likewise, the depolymerization of alkali lignin from wheat straw to liquid oil and decreased solid fraction at 240 °C, 7 MPa and ratio ethanol: water (100:0) is studied [392]. On the other hand, the acid depolymerization of corn stover lignin is proposed using aluminum phosphate zeolite containing Brønsted and Lewis acids at 235 °C for 3 h, to yield monomers (35.7%) and solids (38.15) [39].

Electrochemical processes are developed by applying an electric current to an electrode in order to promote the oxidation of a mediator that acts on lignin generating oxidized lignin products [393]. Studies have shown the generation of sodium levulinate, sodium 4-hydroxyvalerate, sodium acetate and sodium formate by electrochemical depolymerization (100 mA over 8 h) using γ-valerolactone, and ${\mathrm{Na}}_{2}{\mathrm{CO}}_{3}$ as electrolyte [394]. One method in recent research is focused on the use of ionic liquids as solvents, acting as catalysts in order to improve the selectivity of depolymerization. Dier et al. [395] employ ionic liquids such as 1-ethyl-3-methylimidazolium, trifluoromethanesulfonate and triethylammonium methanesulfonate to depolymerize alkali and organosolv lignin for high-value chemical obtainment.

With respect to pyrolysis, it is performed at temperatures around 500 °C for biomass depolymerization [380]. Specifically, lignin from LAIWs such as black liquor, corncob, organosolv lignin and sugarcane bagasse organosolv lignin, at temperatures in the range 400–800 °C, render guaiacol derivatives, phenol derivatives, naphthalene, indene, benzene derivatives and syringol as the main chemical compounds in the bio-oil fractions. Furthermore, the pyrolysis of DES–lignin from empty fruit bunches yields phenol at 650 °C, while lignin pyrolysis from ionic liquid treatments on empty fruit bunches, sugarcane straw and industrial lignin produces chemicals such as formaldehyde, phenol, methanol, naphthalene, alkanes and phenolics. The pyrolysis of kraft lignin, due to its high sulfur content, is a major disadvantage as it causes environmental pollution and, in addition, the oils obtained are very acidic, so kraft lignin must be purified prior to depolymerization [354]. These authors also reveal that, in general, an increase in the molecular weight of lignin will result in lower bio-oil yields, and an increase in the solid and liquid fractions during pyrolysis. One of the most important applications of the pyrolysis process of lignin is to obtain a phenol-rich bio-oil. In this line, the pyrolysis process of lignin from the palm kernel shell is performed to generate bio-oil with a high fraction of phenolic (at 400 °C) compounds and phenols (at 500 °C) [128]. If the pyrolysis oil needs to be upgraded, some catalytic methods (hydrodeoxygenation, zeolite cracking and hydrogenation) can also be used. One of the most recent techniques for the separation of bio-oil components is the extraction with supercritical fluids, at low temperatures to preserve the nature of the components of the mixture, although column chromatography, solvent extraction (dichloromethane, diethyl ether, water, supercritical ${\mathrm{CO}}_{2}$, ethyl acetate, toluene, n-hexane, etc.) and vacuum distillation are also researched [396,397]. On the other hand, microwave-assisted pyrolysis at 550 °C catalyzed by activated carbon has been used to obtain phenols and fuels from alkali lignin [398].

Hydrogenolysis treatment emerges as a particularly promising reductive technique to obtain functionalized lignin-based aromatic biomolecules, using frequently mild conditions for lignin depolymerization (temperatures and pressures lower than 320 °C and 30 bars, respectively) to break β-O-4 and α-O-4 bonds, and both metal-based catalysts (as process is frequently catalyzed) and hydrogen or hydrogen source reagents (methanol, ethanol, isopropanol, formic acid, etc.) although other solvents (sodium formate, decalin, tetralin and formic acid) or even lignin itself are being explored as hydrogen donors in the hydrogenolysis of lignin [23,170,348,381,399]. Hydrogen is added to the carbon–carbon and carbon–oxygen bonds of the lignin structure, causing the polymer chains to break and, in this way, smaller molecules are formed. During hydrogenolysis treatment, additional reductive processes such as hydrodeoxygenation (HDO) and hydrogenation reactions can occur [381]. Apart from that, monophenyl products are generally produced from lignin, which may undergo undesired repolymerization reactions and total hydrogenation of the aromatic rings might be avoided [348].

The gasification of lignin involves partial thermal oxidation, using oxidizing agents (oxygen, air, water) in the form of steam. This process will produce a solid phase (ash), a liquid phase (bio-oil) and a gaseous phase (syngas) consisting of $H_{2}$, CO, ${\mathrm{CO}}_{2}$, small chains of gaseous hydrocarbons and solid particles (char or coke). A higher percentage of CO and $H_{2}$ in the gas will be obtained if the lignin–oxidant ratio is higher. Temperatures employed in lignin oxidation when using air, or controlled mixtures of $O_{2}$ and $N_{2}$, fluctuate in the range 500–1000 °C. Catalysts such as dolomite, sodium carbonate, lime and olivine have been used in some lignin gasification studies [400]. In the case of water, as oxidant, temperatures from 300 °C to 1500 °C and $K_{2}{\mathrm{CO}}_{3}$, Ni/Al or Ru/C mixtures are employed. However, when gasification is carried out from the lignin pool no catalysts are used and temperatures between 400 °C and 750 °C are considered [400].

Liquefaction processes are used to depolymerize alkali lignin at 180 °C using catalyzed (CaO/CeO_2_ and CaO/ZrO_2_) methanol and ethanol liquefaction, yielding a high bio-oil yield (50%) [401].

Finally, the biological depolymerization of lignin can be performed by microorganisms capable of producing enzymes such as lignin peroxide, manganese peroxidase, laccase and multifunctional peroxidase [402]. Therefore, lignin-decomposing species comprise mainly white-rot fungi, that directly attack lignin, brown rot fungi, that secondarily promote lignin demethylation as a consequence of primary cellulose attack, and soft rot fungi (obtaining lower lignin degradation compared to white fungus) or bacteria (belonging mainly to three different classes: Actinomycetes (*Streptomyces viridosporus*, *S. paucinobilis* and *Rhodococcus jostii*), α-Proteobacteria (*Brucella*, *Ochrobactrum*, *Sphingobium* and *Sphingomonas*) and γ-Proteobacteria or ligninolytic bacteria (*Pseudomonas fluorescens*, *Enterobacter lignolyticus* or *Escherichia coli*) [403]. Concerning examples of different applications of biological depolymerization, laccasa from white-rot fungus (*Schizophyllum commune*) has the ability to degrade lignin from wheat, maize and sorghum straws, in order to increase its digestibility in ruminants [404]. Additionally, white-rot and brown-rot fungi are capable of depolymerizing lignin to produce different phenolics such as ferulic and vanillic acids and syringyl alcohol [259]. Some bacteria have also demonstrated their ability to deconstruct lignin for lipid production [13].

## 4. Characterization of Recovered Lignin

### 4.1. Lignin Characterization Methods

To determine the effectiveness of the lignin extraction from lignocellulosic biomass during the delignification process, two types of procedures are used. The calculation of the percentage of Klason lignin that remains in the filtered solid (pulp) after the delignification treatment is shown in Equation (3). On the other hand, the percentage of lignin recovered in the liquid after delignification (lignin pool) is calculated in relation to the total content of this biopolymer present in the original biomass (Equation (4)).

(3) $$\%\;\mathrm{delignification}\;=\;\frac{\%\;Lig_{k}^{b}\;m_{b}\;-\;\%\;Lig_{k}^{pu}\;m_{pulp}}{\%\;Lig_{k}^{b}\;m_{b}}\;100$$

(4) $$\%\;\mathrm{delignification}\;=\;\frac{m_{l}^{p}}{\%\;Lig_{k}^{b}\;m_{b}}\;100$$

where $\%\;Lig_{k}^{b}$ is the percentage of Klason lignin in the original biomass; $\%\;Lig_{k}^{pu}$ is the percentage of Klason lignin in the filtered solid (pulp) after the delignification process; $m_{l}^{p}$ is the mass of lignin in the liquid after the delignification treatment; and $m_{bs}$ is the mass of the original residue.

The amount of lignin dissolved, due to the action of depolymerizing agents, can be calculated according to Equation (5).

(5) $$\%\;\mathrm{depolymerization}\;\mathrm{yields}\;=\;\frac{m_{l}^{dep}}{m_{l}^{po}}\;100$$

where $m_{l}^{dep}$ is the mass of lignin dissolved in the solvent used for the depolymerization treatment and $m_{l}^{po}$ is the mass of lignin recovered after the delignification process.

Lignin characterization requires a multimodal approach, employing a combination of spectroscopic, chromatographic, thermal and imaging techniques. Each method offers unique insights into distinct aspects of lignin’s structure and composition, thereby facilitating its potential applications in sustainable materials and biofuels. The selection of a specific technique is often determined by the targeted properties under investigation and the available analytical resources [405].

Over the past few decades, both destructive and non-destructive techniques have been developed to characterize lignin [237]. Destructive methods, such as acidolysis, hydrogenolysis and oxidation, offer insights into specific structural features, particularly β-O-4 linkages [406], while thermal and mechanical properties can be investigated using differential scanning calorimetry (DSC) and thermogravimetric analysis (TGA) [407]. In contrast, non-destructive methods, primarily spectroscopic techniques, provide a more holistic view of lignin’s structure [408]. Techniques like UV-Vis, Fourier Transform Infrared (FTIR) spectroscopy, Raman and NMR spectroscopy offer detailed information on functional groups, bond types and overall molecular architecture. Advanced NMR techniques, such as two-dimensional heteronuclear single-quantum coherence spectroscopy (2D-HSQC) and ^31^P NMR, are particularly powerful in characterizing isolated lignin fractions, providing insights into the distribution of different structural units and the extent of modification [409].

#### 4.1.1. Differential Scanning Calorimetry (DSC)

This technique is widely utilized for analyzing the thermal properties of lignin, providing valuable information about its glass transition temperature (Tg), thermal stability and other phase transitions. Tg correlates with the molecular weight of polymers and lignins, as well as other molecular characteristics such as branching, cross-linking and molecular flexibility. Tg is also influenced by carbohydrate contamination [410]. This method is essential for understanding the behavior of lignin in various applications, particularly in materials science. DSC is primarily used to determine the Tg of lignin, which indicates the temperature range where lignin transitions from a rigid or glassy state to a more flexible rubbery state. DSC is performed under a nitrogen atmosphere using a heating rate of 10–20 °C/min. The Tg values may vary with the type of lignins deriving from the treatment and biomass source, such as soda lignin from flax (138 °C), kraft lignin from elm (137 °C), organosolv lignin from black locust (155 °C) and soda lignin from orange trees (100 °C) or olive trees (126 °C). Lignins with higher Tg values generally exhibit greater thermal stability. The increased stiffness and reduced mobility of molecular chains contribute to enhanced resistance to thermal degradation. For instance, lignin types with Tg values around 160 °C or higher tend to show improved thermal stability compared to those with lower Tg values [411]. Furthermore, lignin structure, characterized by a high degree of aromaticity and hydrogen bonding, contributes to its elevated Tg. These structural features enhance intermolecular interactions, which in turn improve thermal stability. For example, the presence of guaiacyl units in softwood lignin results in higher thermal stability compared to hardwood lignins with more syringyl units.

#### 4.1.2. Thermogravimetric Analysis (TGA)

It is a crucial analytical technique used to characterize lignin. This method provides insights into the thermal stability, composition and degradation behavior of lignin, which are essential for understanding its potential applications in various industries, including biofuels and materials science. TGA measures the weight loss of a sample as it is heated at a controlled rate. The applications of TGA in lignin characterization are ranging from thermal stability assessment and decomposition behavior in order to compare the various lignin types [412]. In particular, samples are investigated under oxygen or nitrogen atmosphere with a heating rate of 10–20 °C/min and heated from 20–900 °C. This analysis of the first derivative of the TGA (DTG) allows researcher to determine the onset temperature of decomposition and the maximum degradation rate comparing various lignins. The thermal degradation of lignin occurs in distinct stages, typically characterized by weight loss at specific temperature ranges. For example, peaks around 100 °C are typically due to the water content in lignin, while weight loss peaks at 305–350 °C and 600–740 °C are caused by dehydration in lignin and the second weight loss is due to the thermo-oxidative degradation of lignin macromolecular chains [413]. Lignin is a complex, heterogeneous polymer, and its thermal degradation can involve multiple overlapping reactions, making it challenging to precisely interpret all the features in the TGA curve. For this reason, TGA is often combined with FTIR to provide a comprehensive understanding of the gases evolved during thermal decomposition. This combination allows the identification of specific functional groups and the characterization of gaseous products [412].

#### 4.1.3. X-Ray Diffraction (XRD)

X-ray diffraction (XRD) is a non-destructive technique for characterizing lignin, primarily to assess its degree of crystallinity and structural order. The way the X-rays interact with the atoms in the sample through diffraction provides information about the arrangement of those atoms. Crystalline materials have a highly ordered, repeating arrangement of atoms, leading to sharp and distinct peaks in the XRD pattern. Amorphous materials, like many lignin samples, exhibit broad, diffuse peaks or even a halo in the XRD pattern, indicating a lack of long-range order [414]. While not as detailed as techniques like NMR, XRD can provide some insights into the arrangement of lignin molecules, such as the presence of ordered domains or the formation of specific crystalline phases (though rare in lignin). XRD can be used to study the structure of lignin-based materials, such as lignin-based polymers, composites and carbon materials. It can help assess the degree of dispersion of lignin within the matrix, the formation of new crystalline phases and the impact of processing on the material structure [415]. XRD is more sensitive to crystalline regions. Amorphous lignin often produces weak or broad diffraction signals, making it challenging to obtain detailed structural information [416].

#### 4.1.4. Size-Exclusion Chromatography (SEC)

Size-exclusion chromatography (SEC) is a technique for characterizing lignin, used for determining its molecular weight distribution. The technique allows the determination of molar masses, especially weight average molar mass and the average molar mass, helping to characterize lignin’s heterogeneity. Studies have shown that different lignin extraction methods yield fractions with varying molecular weight [417]. Lignins are typically analyzed by alkaline SEC using serial connected columns at 30 °C with 1 mL/min 0.5 M NaOH, performing detection at 280 nm. Measurements are relative to polystyrene sulfonate as standard [418]. SEC is often combined with ultracentrifugation to better compare with the absolute values of weight average molar mass determined with this technique [419].

#### 4.1.5. UV-Vis Spectroscopy

UV-Vis spectroscopy is a valuable tool for characterizing lignin, particularly acid-soluble lignin. The National Renewable Energy Laboratory (NREL) has developed a standardized method for accurately determining lignin content by measuring absorbance at specific wavelengths [183]. Furthermore, lignin’s UV spectrum can display characteristic absorption bands at approximately 200, 240, 280 and 320 nm, directly linked to its inherent molecular structure. Going down to details, the peak at 200 nm corresponds to $\pi \to \pi^{*}$ transitions in aromatic rings, while peaks around 240 and 282 nm are associated with free and etherified hydroxyl groups. The absorption band near 320 nm is attributed to $\pi \to \pi^{*}$ transitions in lignin units with C_*α*_-C_*β*_ linkages conjugated to aromatic rings and n$\to \pi^{*}$ transitions in units containing $C_{\alpha }$=O groups. In herbaceous plants, the presence of bound hydroxycinnamic acids, particularly p-coumaric and ferulic acid, can contribute to this absorption [420]. Quantitative determination of lignin through UV-Vis often suffers due to containing interfering peaks from carbohydrate degradation that reduces molar absorptivity, ϵ, in applying Lambert–Beer equations. Absorptivity constants for acid-soluble lignin measurement were standardized and proposed for selected biomass, as well as specific wavelengths to reduce the aforementioned effect [183].

#### 4.1.6. Fourier Transform Infrared (FTIR) Spectroscopy

Fourier Transform Infrared (FTIR) spectroscopy plays a crucial role in characterizing lignin by providing detailed information about its structural and functional properties. FTIR can be used to analyze lignins in both solid and liquid states, with liquid analysis being particularly useful for rapid process control in pulping. FTIR spectroscopy is a widely used, non-destructive and rapid technique for identifying functional groups in lignin. In particular, mid-infrared spectroscopy (4000–200 ${\mathrm{cm}}^{-1}$) is a reliable technique for analyzing the chemical composition of lignin fractions or wood-based materials [421]. It can detect key functional groups like hydroxyls, carbonyls, methoxyls, carboxyls, and both aromatic and aliphatic C-H bonds.

FTIR spectroscopy provides valuable insights into the structural features of lignin. Key spectral regions and their corresponding functional groups are as follows:

- OH and CH Stretching Region (3460–2800 ${\mathrm{cm}}^{-1}$): O-H stretching of phenolic and aliphatic structures (3410–3460 ${\mathrm{cm}}^{-1}$) takes place, while C-H stretching is found in aromatic methoxyls, methyl and methylene groups (2938, 2842 ${\mathrm{cm}}^{-1}$). A further example is additional aliphatic methylene bands in hemp, jute and flax lignins (2917, 2847 ${\mathrm{cm}}^{-1}$), that can be originated from fatty acids in lignins.
- Carbonyl/Carboxyl Region (1750–1600 ${\mathrm{cm}}^{-1}$): Weak bands that may be attributed to unconjugated carbonyl/carboxyl stretching (1705–1720 ${\mathrm{cm}}^{-1}$) are found, with shoulder peaks attributed to conjugated carbonyl/carboxyl stretching (1680 ${\mathrm{cm}}^{-1}$). Intensity variations and broadening at 1705–1600 ${\mathrm{cm}}^{-1}$ in oxidized, solvent-extracted lignins, sulfur-free lignin depending on the treatment may be originated due to potential protein or water-related bands around 1650 ${\mathrm{cm}}^{-1}$.
- Aromatic Region (1600–1400 ${\mathrm{cm}}^{-1}$): There are characteristic lignin absorption peaks at 1508 ${\mathrm{cm}}^{-1}$ and 1595 ${\mathrm{cm}}^{-1}$ [422,423]. Aromatic skeletal vibrations (1600, 1515, 1426 ${\mathrm{cm}}^{-1}$), C-H deformation and aromatic ring vibration (1462 ${\mathrm{cm}}^{-1}$) can be detected as variations in intensity based on S/G ratio.
- Fingerprint Region (1400–400 ${\mathrm{cm}}^{-1}$): Complex region difficult to analyze due to band complexity with contributions of various vibration modes. This region provides information about monolignol units and linkages, including guaiacyl unit bands (1269 ${\mathrm{cm}}^{-1}$, G ring and CO stretch; 1140 ${\mathrm{cm}}^{-1}$, CH in-plane deformation; 854 and 817 ${\mathrm{cm}}^{-1}$, CH out-of-plane vibrations in position 2, 5 and 6 of guaiacyl units). In this region can also be found information on syringyl unit bands (1326, 843 ${\mathrm{cm}}^{-1}$), that are typical of hardwood and all non-wood lignins e.g., wheat straw, acacia and eucalyptus. Lastly, phenolic OH and aliphatic C-H bands (1370–1375, 1215–1220 ${\mathrm{cm}}^{-1}$), and potential polysaccharide-related bands (1000–1300 ${\mathrm{cm}}^{-1}$) can also be detected.

#### 4.1.7. Raman Spectroscopy

Despite being a well-established technique, Raman spectroscopy’s application to lignin and lignin-containing materials only emerged in the early 1980s. This delay can be attributed to several factors such as limited user familiarity, the perceived redundancy with IR spectroscopy, even if the two techniques are actually complementary, and the high cost of Raman instrumentation, but also to the lignin auto-fluorescence induced by Raman visible range excitation. Over the last few years, various Raman spectroscopy sub-techniques, including visible-Raman, micro-Raman, FT-Raman, Raman imaging, UV-resonance Raman, surface-enhanced Raman and coherent anti-Stokes Raman spectroscopies, have been applied to study lignin and lignin-containing materials [424]. Particular interest was given to FT-Raman, due to its ability to avoid the auto-fluorescence effect. FT-Raman has characteristic regions in the 2700–3200 ${\mathrm{cm}}^{-1}$, 1350–1850 ${\mathrm{cm}}^{-1}$ and 250–1450 ${\mathrm{cm}}^{-1}$ ranges, which assignment is summarized as follows:

- Aliphatic and Aromatic C-H Stretching: Bands in the 2700–3200 ${\mathrm{cm}}^{-1}$ region indicate C-H stretching vibrations in aliphatic and aromatic groups. The intensity of the 2940 ${\mathrm{cm}}^{-1}$ band, associated with methoxyl groups, may be higher in hardwood due to its higher syringyl content.
- Aromatic Ring Vibrations and Carbonyl/Carboxyl Stretching: The 1350–1850 ${\mathrm{cm}}^{-1}$ region shows bands related to aromatic ring vibrations, C=C and C=O stretching. Differences in band intensities can be found between softwood and hardwood due to variations in aromatic ring-conjugated structures [425].
- Fingerprint Region: The 250–1450 ${\mathrm{cm}}^{-1}$ region reveals differences between softwood and hardwood, with certain bands being more intense in one type or the other. These differences can be attributed to variations in specific functional groups and linkages.

Overall, Raman spectroscopy provides valuable insights into the structural differences between softwood and hardwood lignins. By analyzing the intensity and position of specific bands, information can be obtained on the relative abundance of different functional groups and the extent of conjugation in the lignin structure [426].

#### 4.1.8. Nuclear Magnetic Resonance (NMR) Spectroscopy

Compared with the spectroscopic methods mentioned above, NMR spectroscopy offers significantly higher resolution and provides a more comprehensive understanding of lignin structure. It enables the precise quantification of functional groups, linkages and the relative abundance of H, G and S units. Various NMR techniques, including 1D, ^1^H, ^13^C, ^19^F [427], and ^31^P NMR and various 2D NMR, can be applied to both solid and liquid lignin samples. This allows for a detailed analysis of lignin’s composition and structure, qualitatively and quantitatively [405].

^1^H NMR is probably the fastest and cheapest NMR technique to approach the chemical structure of lignins. Analysis can be performed in acetylated or non-acetylated samples. Acetylation of lignin samples simplifies the NMR spectrum by generating derivatives with broad proton signals and minimal interference. This process also distinguishes between aromatic and aliphatic acetyl groups. In contrast, non-acetylated lignin spectra, even if similar to acetylated ones, lack the acetate signals. They exhibit a phenolic proton signal, but aliphatic hydroxyl signals are not well distinguished due to overlap. Non-acetylated lignin spectra show less information, but are not affected by the formation of derivatives [428].

^13^C NMR spectroscopy enables the identification of carbon atoms in various structural and chemical environments within lignin. By quantifying these carbon signals, key structural parameters such as the degree of condensation, the syringyl:guaiacyl:p-hydroxyphenyl (S:G:H) ratio and the predominant linkages can be determined [429]. This spectroscopy offers higher resolution compared to ^1^H NMR, allowing the differentiation of overlapping signals in complex lignin samples. However, to obtain accurate and reliable results, it is crucial to use relatively pure lignin samples to avoid spectral interference from impurities [430]. To assign NMR peaks, there are literature examples and databases.

^31^P NMR spectroscopy is a powerful technique for the classification and quantification of various hydroxyl groups in lignin. It employs a phosphitylating reagent (2-chloro-4,4,5,5-tetramethyl-1,3,2-dioxaphospholane) to differentiate between phenolic condensed units such as DPM, diaryl ethers, biphenolics and carboxylic acids [431]. ^31^P NMR offers significant advantages over ^13^C ^1^H NMR for lignin characterization, particularly in terms of specificity for hydroxyl groups, sensitivity due to high natural abundance, rapid analysis capabilities, clear chemical shift patterns and reduced complexity in spectral interpretation [432].

Two-dimensional NMR techniques offer higher resolution than 1D NMR, enabling more accurate identification and assignment of signals, particularly in complex lignin structures overcoming the overlapping of resonances [405]. Two-dimensional NMR methods, such as heteronuclear multiple-quantum coherence (HMQC) spectroscopy, heteronuclear correlation (HETCOR) spectroscopy, homonuclear Hartmann–Hahn (HOHAHA) spectroscopy, total correlation spectroscopy (TOCSY), rotating frame Overhauser experiment spectroscopy (ROESY), heteronuclear single-quantum coherence (HSQC) spectroscopy and heteronuclear multiple bond coherence (HMBC) spectroscopy, have been employed in lignin structure characterization. Among these techniques, 2D HSQC NMR is the most widely used due to its ability to effectively illustrate structural features and transformations in isolated lignin fractions. For example, this technique can directly characterize the structures of lignin and polysaccharides within intact cell walls, including the linkages between them, without requiring the prior isolation of individual components. Finally, combining quantitative ^13^C NMR with 2D HSQC NMR is a powerful approach for elucidating the structure of complex samples. By using the 2D spectrum as an internal standard, this method can address issues like signal overlap and quantification errors associated with different line widths and the presence of various structural units [433].

#### 4.1.9. X-Ray Photoelectron Spectroscopy (XPS)

X-ray photoelectron spectroscopy (XPS) is a surface analysis technique that provides information about the elemental composition and chemical state of a material’s surface. By bombarding a sample with X-rays, electrons are ejected from the atom surface. The binding energy of these emitted electrons is characteristic of specific elements and their chemical environment [434]. XPS can be used to identify the elements present on a surface, quantify their relative abundance, and determine the types of chemical bonds formed by those elements. For example, in the case of lignin, XPS can be used to analyze the carbon, oxygen, nitrogen, sodium and sulfur content. High-resolution XPS can further differentiate between different carbon bonding environments, such as C-C, C-O and C=O [407]. Despite XPS being a powerful technique, it has limitations. It cannot detect hydrogen atoms, which can make it difficult to distinguish between certain functional groups. However, XPS is a relatively simple, non-destructive technique that has been widely used to characterize lignin and its modifications. It has been employed to study the effects of plasma treatments, enzyme adsorption and other surface modifications on lignin properties [435].

## 5. Industrial Applications of Lignin

Using lignin derived from LAIWs (walnut shell or corn stalk [436]), through green processes in harmony with the environment, is one of the current emerging lines of research [437]. The lignin recovered from LAIWs (technical or industrial lignin) could be structurally modified, with respect to the lignin present in the original biomass (natural lignin), depending on the treatment selected for its extraction. For this reason, not all the recovered lignins are suitable for every application, as their characteristics are closely linked to their chemical structure, so potential applications are conditional upon their properties. For example, while lignosulfonates are fully soluble in water, kraft and soda lignins will be soluble in aqueous solvent only under alkaline conditions. On the other hand, alkali and organosolv lignins will be soluble in a wide range of organic solvents, apart from water (pH > 12) [175,438].

Thus, when a lignin with high antioxidant power is the purpose, it is better to use treatments that provoke a greater rupture of the ether bonds, favoring the presence of greater quantities of phenolic hydroxyl groups that contribute to the increase in its antioxidant capacity, and also increase its thermal stability. On the other hand, if lignins preserving their initial integrity are required for a specific application, it is better not to use methods such as organosolv, kraft or soda pulping as the lignin obtained is very condensed, with few β-O-4 bonds that can be broken, decreasing the degree of polymerization in the lignin. This would prevent the use of these lignins in the production of small molecules, as there would be no feasible bonds left to be broken [439].

Therefore, kraft lignin is heavily modified by the breaking of β-ether (C-O) bonds and removal of methyl groups during the process [175]. It also has a high degree of condensation because strong alkaline conditions generate reactive ionized groups that can participate in condensation reactions. High temperatures can also accelerate these reactions, and sodium sulfide can react with the lignin to form lignosulfonates that can undergo further condensation reactions. Some industrial applications of kraft lignin as resins, pesticides, fertilizers, binder and aromatic chemicals are restricted due to a high content of ash (0.5–3.0%), sulfur (1–3%) and carbohydrates (1.0–2.3%) [175]. In other cases, the extracted kraft lignin can have annexed hemicellulose-related residues, leading to lignins with amphiphilic behavior like surfactants [177]. Soda lignin, although containing small amounts of minerals (Na and K), carbohydrates and free phenolic groups (by breaking α- and β-aryl ether bonds), has acceptable purity (80%) and it is free of sulfur, so its main applications are focused on phenolic resins production, animal nutrition, dispersants and polymer synthesis [175]. The high molecular weight of sulfite lignin, due to the condensation of aromatic rings, and its high solubility in water focus its main exploitation in the manufacture of adhesives. Sulfite lignin can form polyelectrolytes because they contain ionizable functional groups (sulfonic, carboxyl and phenolic hydroxyls) when dissolved in water, giving rise to a molecule with multiple negative electrical charges. The electrostatic repulsion between negative charges of the sulfonate groups causes the lignin chain to expand, occupying a larger volume in solution, also enhancing the solubility of the lignin in water through ion–dipole interactions. Furthermore, polyelectrolytes could interact with cations to give rise to chemical complexes that modify the solution properties. In the case of organosolv lignin, it has a very low level of impurities (ash and carbohydrates), which greatly broadens its field of application.

Apart from the degree of polymerization of the precipitated lignin, another important aspect is its coloring, a key property in certain commercial applications, because lignins recovered from pools usually exhibit a dark brown or black color. Some factors that may influence the lignin color are the presence of chromophore groups (i.e., quinones and methoxy substituted phenoxy groups, conjugated with double bonds or carbonyl groups), low pH values needed to precipitate lignin or the drying temperatures used [334]. For example, lignins precipitated at pH = 2 have a brighter color than those at slightly higher pH values (3–5). In some applications, the dark color of lignins can be beneficial (dyes in the textile industry, paints and coatings, pigmented biodegradable plastics, dark concrete, asphalt, etc.) but, in other cases, this coloring must be masked as is the case when lignin is used as an integral part of transparent films [334]. Concerning masking procedures, bleaching, chemical alterations of lignin structure and encapsulation of the biopolymer to hide its color, as well as mixing with other compounds to lighten its coloring could be useful techniques.

**Figure 4:**
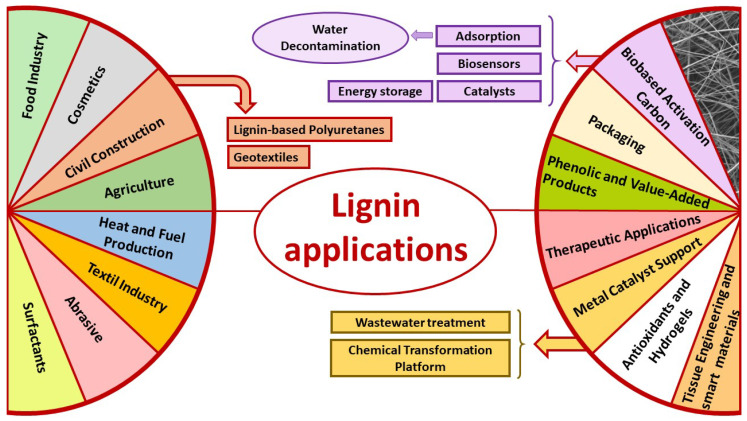
Main industrial applications of lignin.

In general, lignin has many industrial applications as it is a natural biopolymer with high oxidation resistance, high thermal stability and calorific value, and high robustness [437]. For lignin harnessing, so moving away from traditional biomass incineration activities with high emissions of greenhouse gasses, in Figure 4 several possibilities are proposed.

1. Heat and fuel production: Bio-oil obtained by pyrolysis, gasification or hydrothermal liquefaction can be used for heat production by direct burning. However, bio-oil contains significant amounts of water, acids and heavy oligomers, so it has a low energy content that makes it unviable as a vehicle fuel, unless it is subjected to hydrodeoxygenation processes [397]. Additionally, steam reforming of pyrolysis bio-oil from biomass is intensively being investigated to produce hydrogen. Apart from that, lignin pyrolysis processes give rise to significant amounts of phenols and phenolic compounds, depending on the experimental conditions employed. These phenolic compounds can form part of phenolic resins that are highly valued as binders for composite wood products, such as plywood and oriented strand boards. Another possible way of utilizing lignin would be through combustion processes, for example of waste black liquor from kraft pulp production [440], generating ${\mathrm{CO}}_{2}$ and $H_{2}$O as the main products, which can be used to produce heat or electricity. Additionally, lignin can be also converted into suitable diesel, gasoline and aviation fuel [441]. Another study demonstrates that lignin from wheat straw can be converted, by catalytic depolymerization, into aromatic C6–C8 fractions and can subsequently give rise to aromatic C8–C15 fractions, by low-temperature alkylation using ionic liquids, with suitable characteristics for jet fuels [442]. Other studies focus on the harnessing of lignin from LAIWs (e.g., wheat straw) by different gasification technologies (fixed bed updraft, bubbling fluidized bed and indirect gasification) for the production of syngas that can be fermented to obtain bioalcohols [443].
  The techno-economic assessment of the application of lignin to heat and fuel production (e.g., by gasification or hydrothermal liquefaction) shows significant incentives such as those derived from life cycle analysis or those associated with the price of fossil fuels [444].
2. Civil construction: Lignin is added to cements and mortars, to make lignin-based cement composites. In this way, the amount of water required is reduced, increasing the particle-to-particle adhesion, plasticity and workability. Lignin may adhere to the particles and act as dispersant, providing steric hindrance and electrostatic repulsions, that prevent the formation of aggregates when lignin is adsorbed or adhered on the cement particles through the formation of a film [445,446]. Accordingly, lignin-based polyurethanes can be produced by replacing polyols from urethane with lignin, originating composites with modified properties with applicability as foam, for thermal insulation, to improve the energy efficiency of buildings apart from gap filler and adhesive for materials such as wood, metal, concrete and ceramics [447]. It also has been shown that lignin molecules can adhere to the surface of the metal, forming a protective layer with an anti-corrosion effect [445]. These protective properties in coatings and adhesive properties have also been demonstrated in lignin-based epoxy resins [132]. Additionally, lignin-based phenol resins are utilized in the production of various building materials such as particleboards, laminates, coatings, plywood and adhesives.
  There are also studies that demonstrate the benefits of lignin in the field of pavement engineering, as lignin-modified asphalt has advantages over conventional asphalt in terms of improved properties of the resulting material [448]. In this sense, the addition of lignin can not only reduce the proportion of asphalt in the asphalt pavement, but also improve its properties. Moreover, from an economic point of view, the use of lignin in the asphalt binder industry can save costs, given the market price of lignin (about USD 297 per ton) compared to that of asphalt (USD 484 per ton), positioning lignin pavement as a promising material in upcoming applications [448]. In addition, lignin can also act as a protective coating of natural fiber geotextiles against degradation processes in materials [449]. The incorporation of lignin in geotextiles used in pavements can increase their durability and wear resistance, preventing blockages. Geotextiles containing lignin can also protect underground pipelines from damage caused by soil movement.
3. Bio-based activated carbon attainment: Composite carbon nanofiber from lignin can be obtained considering four sequential stages: fiber spinning, fiber stabilization, fiber carbonization and surface treatment [450]. In particular, this promising porous material could be employed for energy storage or adsorption. Energy applications are based on capacitors and battery production while adsorption methods can also be conducted, through electrostatic interactions of compounds with the polarized surface of the bioadsorbent, offering advantages such as low-cost, high surface area, high thermal and electrical stability, suitable thermal properties and corrosion resistance [436]. The current reliance on polyacrylonitrile for the production of carbon nanofibers limits and makes their industrial applications more expensive, with a market price in the range of USD 5–44/kg. The incorporation of lignin would reduce these costs, expanding the current demand for carbon fibers (140,000 tonnes in 2020) [451].
  Concerning energy storage, lignin-based phenolic resin carbon microspheres have been used to produce capacitors [452]. Related to sustainable energy storage, the generation of hydrogels from lignin, used for the preparation of electrolytes, is seen as a promising alternative for organosolv lignin [453]. The combination of these hydrogels with lignin-derived electrodes results in supercapacitors with improved electrochemical properties. Likewise, activated carbons prepared from waste lignin, once thermochemically activated (microwave-assisted KOH treatment) were integrated together with polyvinyl alcohol to form electrodes in supercapacitors, using LiCl as the gel electrolyte [454]. These authors also obtained, through a carbonization process followed by microwave-assisted chemical activation of lignin, a superconductor with a low production cost.
  These energy applications of activated carbons from LAIWs are not restricted to the production of supercapacitors; empty fruit bunch and rice husk KOH activated carbons have been used to produce biomaterials for hydrogen storage [168] and residual lignin can also be integrated in nanocomposites to form battery anodes and cathodes for hydrogen storage. The large number of oxygen atoms in lignin favors the adsorption of electrolyte ions, and the occurrence of redox reactions, increasing the energy storage capacity in batteries and supercapacitors [437]. Other recent research into the use of lignin includes the use of consistent nanoporous carbon, generated by low-temperature carbonization (350 °C, 30 min) and physical activation with ${\mathrm{CO}}_{2}$ (1000 °C) of organosolv lignin from materials such as hemphurds, eucalyptus chips, flax straw and rice husk for hydrogen storage by adsorption [455].
  On the other hand, hydrocarbons from the hydrothermal carbonization of coconut shell powder, in the presence of zinc chloride, leads to a mesoporous material rich in functional groups suitable for contaminant removal [456]. In the same line, activated carbon, obtained by chemical activation of charred materials, has higher porosity and surface area than that obtained by physical processes. The chemical treatment of lignins to obtain activated carbon involves the use of high temperatures (500–900 °C), inert atmosphere of Ar or $N_{2}$, in the presence of agents such as $H_{3}{\mathrm{PO}}_{4}$, NaOH, $K_{2}{\mathrm{CO}}_{3}$, ${\mathrm{ZnCl}}_{2}$ or preferably KOH as it generates a large number of pores with a high specific surface area [436].
  In this line, porous carbon materials have been applied to water treatment, using for example alkali lignin to retain Congo red effectively by adsorption [457]. An important application of lignin is in the adsorption of chromophore groups present in dyes, through different types of interactions: through the hydrogen bonding of OH groups of lignin and polar functional groups of chromophore dyes (carbonyls or amines). Hydrophobic interactions (London dispersion forces) between aromatic rings of the lignin and hydrophobic portions of chromophore groups could also take place. In addition, ionic interactions of charged lignin groups (sulfonates and carboxyl groups) with charged chromophore groups could also occur.
  Kraft lignin is rich in hydroxyl and aliphatic groups, both polar, so it has a high affinity for polar aromatic groups and some metals. It has been successfully used for the removal of dyes by the formation of kraft lignin hydrogels [458]. With respect to sulfite lignin, it contains a high amount of sulfonate groups, which makes it highly soluble in water, limiting its capacity to retain heavy metals (lead, cadmium and chromium) from wastewater [459], although electrostatic interactions with sulfonate groups and the use of modified lignins could be promising alternatives to improve their adsorptive capacity. Concerning organosolv lignin, it has a more conserved and less condensed structure than other lignins and, in general, tends to be more hydrophobic than kraft or sulfite lignin due to its preserved structure rich in aromatic rings and alkyl groups, which are hydrophobic. Nevertheless, its adsorption capacity depends on the nature of the solvent used in the extraction and the reaction conditions. In general, organosolv lignin has a limited solubility in water, so it has a great potential for the removal of pollutants from water [459]. Finally, it is possible to modify the properties of lignin by oxidation, reduction or functionalization processes to improve its adsorption capacity for certain pollutants. The production of lignin composites is currently a promising alternative for the adsorption of metals in polluted water, for example, by introducing ${\mathrm{SiO}}_{2}$ into the structure of certain LAIWs, such as wheat husks [460]. The structure of lignin, with the presence of a multitude of functional groups, favors interaction with particles dissolved in water, promoting their purification by flocculation processes [446].
  An additional application for lignin-based carbon fiber is the development of biosensors, for the detection and quantification of pathogens [450]. Finally, porous carbonaceous materials derived from lignin as catalysts in ${\mathrm{NaBH}}_{4}$ methanolysis reactions to generate hydrogen should be considered [365].
4. Antioxidants and hydrogels: Significant research efforts have been made in recent years to develop hydrogels with appropriate structures for drug delivery. The variability and complexity of lignin makes it difficult to interact with the three-dimensional matrices of these materials and to create porous scaffolds [461]. The high aromaticity of lignin and, in particular, the presence of phenolic compounds, determine the antioxidant power of lignin. This antioxidant capacity of lignin can be chemically enhanced, resulting in modified lignins. In this context, alkali lignin extracted from cornstalk was chemically modified by the Mannich reaction (using ethylenediamine aqueous solution and, sequentially, formaldehyde), achieving significant increases in their antioxidant activity. This antioxidant capacity could be employed in biomedicine, food packaging, cosmetics and medical devices through the production of lignin-based films as carriers or in biocompatible scaffolds [462]. On the other hand, hydrogels from lignin can be used to encapsulate medicines. The hydrophilic character of lignin allows it to retain water, which causes the three-dimensional network of the lignin to facilitate drug-controlled release at the site of injury [463].
5. Packaging: Lignin-derived biomaterials can be substitutes for plastic, offering more sustainable applications. Thermoplastic and thermosetting lignin polyester has gained great attention [464] to produce flexible and tough lignin-based films for packaging. Regarding this application, oxypropylated kraft lignin (5%) with polylactic acid biocomposites have shown suitable characteristics for being used in food packaging [465]. In addition to antimicrobial and flame retardant properties, the use of lignin to generate UV-blocking materials has also been investigated, attributing this property to the phenolic and carbonyl groups in the lignin [368,466], absorbing light in the UV range (200–400 nm). Apart from that, lignin can also be useful to prepare lignin-based bio-finish in order to provide hydrophobic materials [467]. Transparency of the wrapper is a highly appreciated quality, so the amount of added lignin (which could give a brown coloring) should be optimized. Boarino and Klok [466] collected in their research information on biodegradable polymers based on different types of lignins (soda, kraft, organosolv and lignosulfonates) with different matrices (polylactic acid, polyvinyl alcohol and poly(3-hydroxybutyrate)/polyhydroxyalkanoates), showing how soda lignin biocomposites have antioxidant capabilities and UV barrier activity, only demonstrated for punctual kraft and organosolv lignins. Additionally, from lignin, different phenolic compounds can be obtained to be used in packaging applications. There are microorganisms (*Actynomyces, Aspergillus, Clostridium and Lactobacillus* species) capable of generating feruloyl esterase enzymes, necessary for the generation of phenolic compounds from lignocellulosic biomass. The addition of feruloyl esterase to the biomass (usually pretreated) allows the hydrolysis of the ester bonds, releasing ferulic acids and other phenolic compounds [196], acting as protective coatings on cardboard boxes, paper bags and other types of packaging. Ferulic acid has been used in blends with polymers such as low-density polyethylene and ethylene vinyl acetate to make effective packaging films [468]. Likewise, a recent study [469] was able to effectively incorporate lignin nanoparticles into starch polymeric matrices, obtaining biofilm composites that were used for soybean oil packaging, observing a delay in its oxidative deterioration.
  For food packaging applications, lignin can not only improve the mechanical properties of polymeric biofilms, but also antioxidant and anti-UV barriers. The main challenge is to achieve their compatibility with the surrounding matrix, through proper lignin functionalization. Another challenge is to improve the digestibility of biofilms in composting processes [466].
6. Agriculture: The application of lignin-based materials for agricultural mulching increases the water and nutrient retention capacity of the soil, as well as its organic matter supply through the gradual decomposition of lignin [470]. Lignin can be transformed into agrochemicals such as slow-release nitrogen fertilizer, pesticides, soil amendments and plant growth regulators [471]. The use of lignin-coated urea reduces the rate at which urea dissolves in the soil, reducing soil losses and controlling nitrogen levels, implying a higher utility of mixed lignin compared to the use of urea alone, as it provides higher crop yields [471]. On the other hand, when lignin is added to pesticides, in some cases, lignin can act as a dispersant, reducing the attractive forces between pesticide particles and thus reducing viscosity. As pesticides can penetrate more quickly, increasing the effectiveness and decreasing the consumption of the product. Thus, the use of lignin in the development of innovative materials and formulations, compared to traditional agrochemicals, is one of the main challenges in agriculture, demonstrating a growing use, high efficacy and cost-effectiveness [472].
7. Surfactants: Lignin has great potential as a low-cost surfactant because of its hydrophobic aromatic configuration and multifunctionality. In this sense, lignosulfonates have been used as a surfactant in industry, but their purity and limited efficiency have hindered their application. This justifies the need to advance in the knowledge regarding the performance and property correlation of lignin-based surfactants in order to be widely incorporated in the market [473]. Kraft lignin is an excellent starting material to obtain surfactants with similar behavior to synthetic ones, as it possesses a wide variety of functional groups such as hydroxyl, carbonyl and methoxy which give it an amphipathic character (there are hydrophilic and hydrophobic parts). In this context, lignin from kraft pulping was diluted in ammonia and then soaked in a dilute solution of polyvinyl alcohol for surfactant generation [374]. These surfactants could be used as foamers, dispersants and emulsifiers. Likewise, small concentrations of cationic kraft lignin surfactants successfully stabilized oil–water emulsions [474]. On the other hand, lignosulfonates have been used as anionic surfactants in concrete admixtures, although they have little efficacy in decreasing the surface tension of water or the interfacial tension of water–oil mixtures [473].
8. Biotransformed phenolic and other compounds: Monomers from lignin phenols obtained after the depolymerization of lignocellulosic biomass involves vanillin (producing vanillin alcohol by reduction), vanillic acid (reduced to vanillin), syringic acid that can be reduced to syringaldehyde, guaiacols, (iso) eugenol, ferulic acid, p-coumaric acid and alkylphenols [475], used in food, beverages, cosmetics and pharmaceuticals (due to their anti-inflammatory, antioxidant and anti-tumor properties). The separation of vanillin after the depolymerization (alkaline oxidation) of lignin (Indulin AT) has been efficiently carried out by chromatography, using water and ethanol as eluents [476]. Additionally, pyrolysis, gasification, liquefaction, hydrogenolysis and biological routes are alternatives for the depolymerization of lignin to produce benzene, toluene and xylene. However, the production of these compounds accounts for only 5% of lignin-based chemicals, with the drawback of their low price compared to other chemical compounds such as vanillin [6]. Other researchers have succeeded in obtaining compounds such as sodium levulinate, sodium 4-hydroxyvalerate, sodium acetate and sodium formate from the dearomatization of lignin in aqueous media, using ${\mathrm{Na}}_{2}{\mathrm{CO}}_{3}$ as an electrolyte, by an electrocatalytic process [394].
  Related to phenolic compound production, the most significant cost contributions are solvent consumption and maintenance due to the costs of the dissolution and transformation of lignin to polyphenols. Challenges mostly concern their selling price, as they are heavily dependent on the quality of the lignin-derived resin, which is not currently competitive with those derived from petroleum. The profit could also be increased by reducing costs regarding dissolution, which is a process that is still in its early stage and could have a crucial impact on the product’s final quality [477].
9. Cosmetics: The main applications of lignin in this field are based on its high antioxidant and ultraviolet radiation absorption capacity. There are studies that support the suitability of the use of sugarcane bagasse alkaline lignin in topical applications [373], as ingredient of sunscreens and blemish balm or antiaging cream, temporary skin sensitization, mutagenicity and cytotoxicity tests. The benefits of lignin from hazelnut and walnut or coconut husk, as an ultraviolet radiation protector, have also been corroborated [478,479].
  The global lignin cosmetics market is projected to grow at a compound annual growth rate (CAGR) of 10.7%, reaching approximately USD 13.5 billion by 2032 [480].
10. Textile industry: Lignin is drawn as a textile fiber by the melt spinning method and electrospinning method [481]. The property of lignin to generate char when burnt has been the starting point for the use of this polymer in flame retardants and intumescent polylactic acid textile components [4]. In addition, lignin–silica-based materials recovered from rice husk have also been used as a flame retardant, overcoming one of the most important drawbacks of cotton textiles, namely its high flammability [255]. Some studies reveal that lignin-based epoxy resins can generate highly thermally and mechanically stable materials [132] for creating water, stain and abrasion resistant finishes on a wide range of fabrics. A textile application was established by Abdel-Shakur et al. [482], using corn straw lignin to make single-use medical textiles with demonstrated antimicrobial activity. Lignin can also be used as a dispersant in the textile industry and is expected to grow at an annual rate of 6% between 2020–2026 [483].
11. Food industry: Lignin-derived nanogels, lignin-based films and lignin nanoparticles can also be used in the food field for the delivery of pre-encapsulated vitamins and food dyes [484]. This encapsulation technique using nanoparticles from alkali lignin has also been used to preserve essential oils, increasing their thermal stability as well as their antibacterial properties against *S. aureus* and *E. coli* [485]. Furthermore, lignin–whey protein has been used for the microencapsulation of *Lactobacillus* probiotic bacteria, under similar conditions to those of the digestive system [486]. According to this, alginate microcapsules are frequently used as encapsulation materials although, in order to improve their properties, either alginate microspheres cross-linked with calcium ions or a combination of alginate with other compounds (chitosan, calcium carbonate, gelatin or whey protein) are usually used for better protection of bacterial cells [486].
12. Therapeutic applications: Many monomeric compounds derived from lignin have medical or therapeutic applications: ferulic acid, vanillin, eugenol, syringic acid and coumaric acid. With the exception of coumaric acid, all of them have been shown to have antimicrobial properties. In addition, with the exception of eugenol, they are diabetes-controlling and anticarcinogenic. While syringic acid has anticoagulant properties, eugenol helps control neurodegenerative diseases as do the lignophenols (which also control cardiovascular diseases and diabetes) [487].
  For therapeutic applications, products isolated from lignin or lignocellulosic biomass need to be well characterized to meet regulatory requirements. In this respect, further research is needed on methods for the large-scale isolation of lignin-derived therapeutic products, as well as on the characterization and quantification of products and impurity profiles as a basis for regulatory approval [488].
13. Tissue engineering and smart materials: The use of lignin coatings can promote cell adhesion to certain medical implants, improving their biocompatibility. In this way, the use of chitin–lignin hybrid systems as fillers in polyurethane and foams has been studied [489], including additional properties to polyurethanes such as antimicrobial activity, light weighting and increased environmental sustainability. The field of study of lignin incorporation to promote tissue regeneration is a very novel field that requires further research for the development of lignin-derived biomaterials with suitable properties [461].
  On the other hand, smart materials can also be produced on the basis of lignin. Some examples can be found in the literature [490], in particular in relation to the production of biosensors and shape-programmable materials. In biomedical systems, they are explored for drug and gene delivery, tissue engineering, wound dressing and as pharmaceutical excipients. These materials exhibit properties like antimicrobial behavior, biodegradability, biocompatibility and antioxidant properties.
  These smart materials can act as chemical sensors for detecting metal or chromate ions, formaldehyde or biological species. There are studies with magnetic adsorbents based on functionalized lignin (with an abundance of carboxyl and hydroxyl groups) for the removal of Cr (VI), Cr (III), Cu (II), Zn (II), Ni (II) and Pb (II) [491]. In addition, chitosan smart films containing lignin have been used as biomarkers to monitor food freshness [492]. Concerning biomedical applications, bio-based lignin materials are explored for drug delivery (based on the chemical modification of lignin and polymer grafting strategy), tissue engineering, wound dressing and as pharmaceutical excipients. These materials exhibit properties like antimicrobial behavior, biodegradability, biocompatibility and antioxidant properties. Additionally, as smart coatings, lignin-based graphene avoids the disruption of electron transmission, which is beneficial for use in electromagnetic interference shielding [493]. Finally, shape-programmable materials, due to conductive properties, could detect changes in resistance induced by changes in applied voltage, gaining applicability in human motion sensing [490]. Some main challenges for these smart materials focus on improvements for drug delivery systems, synthesizing materials carrying more than one active substance, with the potential for sequential or simultaneous controlled and site-specific release as well as improvements in the thermo-mechanical properties of these new materials.
14. Metal catalyst support: The wide variety of functional groups in lignin allows it to be used as a support for metal catalysts, as they can be fixed by strong coordination. The main applications of ligninic metal supports are in wastewater treatment, promoting degradation and reduction processes of toxic metals, dyes and phenolic compounds. They are also used in platform chemical transformation (for example in Fischer–Tropsch synthesis to obtain hydrocarbons from syngas, containing CO and $H_{2}$, lignin depolymerization and energy conversion, mainly for hydrogen production. In the latter case, lignin-supported metal catalysts have proven to be effective in hydrogen production processes by water electrolysis or photocatalysis and they accelerate electrocatalysis processes in fuel cells [494].
15. Abrasive: Lignin–alumina hybrid additive results in promising materials for phenolic binders, improving the mechanical properties of certain abrasive materials [495]. Furthermore, some authors [496] conclude that the conditions for obtaining abrasives with the best thermo-mechanical properties involve the incorporation of 5% magnesium lignosulfonate ($H_{2}O_{2}$ oxidized). In relation to advances in sustainable manufacturing, Bellinetto et al. [497] carry out a study expressing the correlation between structure and properties for the case of succinic-anhydride-modified lignin. Moreover, 2% kraft lignin added as an additive to tree-leaf pellets increases the abrasion resistance [498]. Thus, activated lignin can be a very promising renewable and environmentally friendly material for the sustainable development of the modern abrasives industry [496].

## 6. Conclusions

Lignin valorization from LAIWs represents a crucial step toward sustainable industrial practices and a circular bioeconomy. A lignin-based biorefinery approach is mandatory to improve the sustainability of this industry. Future research is required to identify the most appropriate applications of lignin depending on the type of biomass and extraction treatment. Additionally, new horizons in research should focus on the study of treatments that guarantee an efficient separation of the lignocellulosic fractions, thus avoiding lignin condensation. The proposed processes should also address aspects related to biotoxicity, economy and the carbon footprint of the products. This review highlights the advancements in lignin recovery and its potential applications, underscoring the importance of developing efficient, scalable and environmentally friendly extraction methods. Addressing challenges such as lignin structural heterogeneity and the dominance of cellulose-focused research is essential. The ongoing exploration of innovative recovery processes can unlock lignin potential as a high-value co-product, contributing to reduced waste, improved resource efficiency, and enhanced economic and environmental outcomes in the agro-industrial sector.

## Figures and Tables

**Table 1:** Production and composition (dry basis) of the main LAIWs.

LAIWs	Production	Ref. ^(1)^	CEL	HEM	LIG	Ref. ^(2)^
	Mt/year		%	%	%	
Acai seed	1.$4^{\mathrm{Brazil}}$	[33]	3.6	58.0	11.7	[34]
			36.1	16.6	47.9	[35]
Almond hull	1.6	[36]	20–35	10–15	8–15	[37]
Almond shell	2.3	[38]	31–48	27–30	22–32	[38]
Apple pomace	5–7	[39]	47.5	27.8	24.7	[40]
			15.4	12.5	15.5	[41]
Banana peel	36.0	[42]	16.1	10.5	36.3	[43]
Banana leaf	9.3	[44]	43.3	34.3	15.0	[45]
Barley husk	9.3–12.4	[46]	35.6	26.1	14.3	[47]
			39.0	12.0	22.0	[48]
			33.6	37.2	19.3	[49]
Barley straw	51.3	[50]	42.5	26.6	18.3	[51]
			34.3	22.6	20.7	[52]
			38.0	21.9	17.3	[53]
Cashew shell	2.4	[38]	25–35	23–32	14–40	[38]
			40.3	24.8	18.4	[54]
Cassava stalks	32–38	[55]	35.0	21.0	28.0	[56]
			36.3	32.3	19.3	[57]
Cassava peel	27.7–41.5	[58]	43.6	10.4	7.7	[59]
			40.5	21.4	11.7	[60]
Cocoa pod husks	48	[61]	19–35	8–13	14–23	[62]
			23–32	8–38	11–33	[63]
			29–32	25–27	19–22	[64]
Cocoa bean shell	0.7–0.9	[65]	23.4	18.1	29.6	[61]
Coconut husk	7	[9]	35.0	25.8	16.0	[66]
			38.9	30.0	35.0	[67]
Coconut shell	2.4	[68]	33.6	19.3	36.5	[69]
			25.2	27.7	46.0	[70]
Coffee husk	10	[71]	30–35	18–21	10–22	[72]
			39.2	12.6	23.3	[73]
Corncob	200	[74]	17.8	44.7	20.3	[75]
			17.8	44.7	20.3	[76]
Corn stalk	750	[77]	34.0	17.4	16.9	[78]
			38.7	33.6	18.9	[79]
Corn stover	1661	[80]	31.5	18.0	14.1	[81]
			30–40	24–27	17–21	[82]
Corn straw	1.2 × $10^{6}$	[83]	49.7	29.5	12.0	[84]
			35.0	20.0	12.0	[85]
Cotton stalks	90.3–129	[86]	39.4	19.2	23.2	[87]
			40–50	20–30	10–15	[88]
Grape pomace	67.1	[89]	20.9	13.3	34.8	[90]
			19.3	7.2	15.6	[91]
Grape stalk	0.2–0.6	[92]	16.4	18.3	27.8	[37]
			21.7	15.3	29.7	[90]
Hazelnut shell	0.5	[38]	15.4	22.4	25.9	[93]
			17.2	15–28	30–38	[38]
Hemp stalk	0.9		43–47	37–40	20–23	[94]
			34–60	18–37	19–30	[95]
Maize straw	300	[96]	49.7	29.5	12.0	[84]
	1000	[97]	35.9	24.5	20.4	[98]
Melon seed	1.5–2.9	[99]	22.5	27.7	29.9	[100]
Mustard waste	1.$4^{\mathrm{China}}$	[101]	53.2	13.5	1.1	[101]
Oat husk	5.5	[102]	48.4	16.1	16.2	[103]
Oat straw	0.$5^{\mathrm{Mexico}}$	[104]	26–35	25–28	13–16	[105]
			37.6	23.3	12.9	[40]
Oil palm trunk	$18^{\mathrm{Indonesia}}$	[106]	39.4	26.0	6.6	[106]
	$15^{\mathrm{Malaysia}}$	[107]	38–41	12–17	18–23	[108]
Olive pomace	6.0	[109]	9.7	10.9	21.8	[110]
	1.$0^{\mathrm{Andalusia}}$	[111]	10.4	11.5	13.7	[112]
Olive stone	4.1–5.0	[113]	34.2	25.0	33.5	[114]
	0.$4^{\mathrm{Spain}}$	[115]	30.8	17.1	32.6	[116]
Olive-tree leaves	0.8–1.5	[117]	12.4	37.0	17.0	[118]
	0.$4^{\mathrm{Spain}}$		6.5	12.3	25.9	[119]
Olive-tree pruning	34.8	[120]	36.5	21.3	24.1	[120]
			33.9	18.6	23.1	[121]
Orange peel	16	[122]	36.0	10.0	17.0	[123]
			33.3	30.6	12.0	[40]
Orange-tree pruning	18.4	[124]	40.5	29.3	19.0	[124]
	5.$0^{\mathrm{Spain}}$	[125]	49.1	26.9	20.8	[126]
Palm kernel shell	4.6	[127]	41.5	12.4	34.0	[128]
Paper and pulping industry	15	[129]	34.3	20.3	19.3	[130]
Passion fruit peel	0.$50^{\mathrm{Brazil}}$	[131]	48.0	20.8	12.6	[131]
Peanut shell	13.0	[38]	34–43	7–29	22–35	[38]
			36.4	15.6	25.0	[132]
Pigeon pea stalk	10.3	[133]	33.0	24.0	18.3	[134]
			43.2	29.5	15.1	[135]
Pineapple peel	8.3–11.5	[136]	30.4	44.4	4.2	[137]
			24.2	29.4	6.4	[138]
Pine sawdust	0.07	[139]	55.9	15.3	10.6	[87]
			39.4	20.3	31.4	[140]
Pistachio shell	0.51	[141]	38–57	21–31	17–24	[38]
	4–5	[142]	31.2	31.3	21.2	[141]
Rice husk	120	[27]	33.0	24.0	18.0	[143]
			31.3	24.3	14.3	[40]
Rice straw	740	[144]	32–47	19–27	5–12	[145]
Rye straw	8–20	[146]	37.9	36.9	17.6	[103]
Sorghum bagasse	1.$2^{\mathrm{Brazil}}$	[147]	43.6	26.8	26.2	[148]
Soybean husk	18.0–28.7	[149]	29–52	18–34	2–13	[149]
			29–51	10–25	1–18	[150]
Soybean straw	200	[151]	34.0	29.7	17.5	[152]
Spent coffee grounds	6.0	[153]	13.0	42.0	25.0	[40]
Sugarcane bagasse	1.8 × $10^{6}$	[27]	46.2	27.0	23.7	[154]
			30.9	28.2	9.6	[40]
Sunflower stalk	186–8000	[155]	30.8	12.4	19.3	[156]
Tobacco waste	5.8	[157]	30.6	15.0	18.6	[157]
Triticale straw	36.1	[158]	48.5	21.9	11.1	[159]
Walnut shell	2.3	[38]	20–37	15–31	30–52	[38]
Wheat straw	500	[160]	40.2	27.6	21.0	[161]
			27–30	27–50	15–16	[40]

^(1)^ Related to production data. ^(2)^ Related to composition data.

**Table 12:** Experimental conditions using combined technologies for LAIW delignification.

LAIWs	Treatment 1	Treatment 2	Delignification, %	Ref.
Apricot kernel shell	Liquid hot water, 220 °C, 15 min 1:15 LAIW:H_2_O	Soxhlet extraction ethanol or acetone	35–58	[334]
Bagasse	Lactic acid 0.1%, 170 °C, 60 min 1 g LAIW: 10 mL	Lactic acid 50–80% 170 °C, 60 min 1 g LAIW: 10 g acid	69.5–96.6	[98]
Bamboo culms	Hydrothermal, 180 °C, 30 min 10% solid load	Organosolv 30:50:20 Formic acid/acetic acid/water	62.2	[237]
Banana stem	KOH 5%, 90 °C 1 g LAIW: 10 mL	Microwave assisted 25 min, 360 Hz	44.9	[205]
Chestnut shell	Liquid hot water, 100 °C, 24 h 1 g LAIW: 10 mL	Alkaline treatment KOH or NaOH, 1–5%	25-71	[306]
Coconut husk	Steam explosion, 170 °C, 2.5 min 12–15 bar	NaOH 30%, 170 °C, 3 h, 12–15 bar	45.6–55.8	[335]
Corn stalk	$H_{2}O_{2}$ 2%, 80 °C, 120 min 1 g LAIW: 20 mL	Cold plasma 50 °C, 120 min	86	[336]
	$H_{2}O_{2}$ 30%, 80 °C, 120 min 1 g LAIW: 10 mL	Acetic acid, 1:1 *v*/*v*	89	[190]
Corn stover	HCl 1%, 120 °C, 40 min 1 g LAIW: 10 mL HCl	5–26% ${\mathrm{NH}}_{4}$OH, 120 °C, 40 min	40–75	[208]
	Milling, 400 rpm, 1 h	Ethylenediamine (EDA) 120 °C, 60 min	70–85	[337]
Grape stalk	Subcritical water, 170–180 °C 30 min, 1 g LAIW: 10 g	$H_{2}O_{2}$ 4–8%, 40 °C, 1 h 1 g:30 mL	75	[338]
Olive pomace	NaOH 4%, 25°C, 4 h	Microwave assisted 450 W, 10 min	47.1	[213]
Olive-tree pruning	NaOH 0.8%, 60 °C, 59 min	Oxalic acid 0.075 M 150 °C, 30 min, 1 g:10 g	≃20	[339]
	Organosolv	Microwave assisted		[340]
	Acetone 65%	155 °C, 8.4 min	97.3	
	Ethanol 65%	138 °C, 14.8 min	95.5	
	γ-valerolactone 60%	149 °C, 5 min	97.7	
Pistachio shell	NaOH 1 N, 70 min 1 g LAIW: 200 mL	Ultrasound assisted 70 min, 400 W	78.9	[212]
Rice husk	NaOH 4.8% + ${\mathrm{Na}}_{2}{\mathrm{SO}}_{3}$ 3% 1 g LAIW: 12 mL	Microwave assisted 80 °C, 90 min, 400 W	45.5	[341]
	Deep eutectic solvent 120 °C, 1–6 h 1 g LAIW: 10 g DES	Subcritical water 200–260 °C, 15 MPa, 10 min	42–46	[300]
Rice straw	Ionic liquid 1 g LAIW: 20 mL	Sonication assisted 75–90 °C, 80–160 min	22–61	[342]
	$H_{2}{\mathrm{SO}}_{4}$ 3%, 121 °C, 30 min 1 g LAIW: 10 mL	NaOH 4%, 121 °C, 30 min 1 g LAIW:12.5 mL	54.9	[343]
	Liquid ammonia 15% 100–190 °C, 20 min	Acid treatment, 130 °C, 20 min	69–84	[344]
Sisal waste	$H_{2}O_{2}$ ultraviolet cat. 1 g LAIW: 20 g IL	Ionic liquid, 25% TAH 20–100 °C, 20–120 min	79.2	[291]
Sugarcane bagasse	Liquid hot water, 180 °C, 20 min 1 g LAIW: 9 mL	Organosol ${\mathrm{CO}}_{2}$ assisted 140 °C, 45 min	82.5	[309]
Vine shoots	$H_{2}{\mathrm{SO}}_{4}$ 15%, 150 °C	Organosolv 50% ethanol 180 °C, 15% load	43	[345]
Wheat bran	$H_{2}{\mathrm{SO}}_{4}$ 0.05%, 218 °C, 8 min	Hydrotopic solvent 40% *w*/*w*, 1 h	24.1–42.6	[228]
Wheat straw	Autohydrolysis 140–180 °C, 40 min	NaOH 2–6% *w*/*w* 1 g LAIW: 25 g	33.9–65.6	[312]

## Data Availability

Data sharing is not applicable.
